# Investigating the Effects of Chelidonic Acid on Oxidative Stress-Induced Premature Cellular Senescence in Human Skin Fibroblast Cells

**DOI:** 10.3390/life14091070

**Published:** 2024-08-27

**Authors:** Burcu Turkoglu, Banu Mansuroglu

**Affiliations:** Department of Molecular Biology and Genetics, Faculty of Arts and Science, Yildiz Technical University, Istanbul 34220, Turkey; btuncer@yildiz.edu.tr

**Keywords:** chelidonic acid, senescence, senolytic, senomorphic, oxidative stress, H_2_O_2_, premature senescence, BJ Fibroblasts

## Abstract

This study investigated the effects of chelidonic acid (CA) on hydrogen peroxide (H_2_O_2_) induced cellular senescence in human skin fibroblast cells (BJ). Cellular senescence is a critical mechanism that is linked to age-related diseases and chronic conditions. CA, a γ-pyrone compound known for its broad pharmacological activity, was assessed for its potential to mitigate oxidative stress and alter senescence markers. A stress-induced premature senescence (SIPS) model was designed in BJ fibroblast cells using the oxidative stress agent H_2_O_2_. After this treatment, cells were treated with CA, and the potential effect of CA on senescence was evaluated using senescence-related β-galactosidase, 4′,6-diamino-2-phenylindole (DAPI), acridine-orange staining (AO), comet assay, molecular docking assays, gene expression, and protein analysis. These results demonstrate that CA effectively reduces senescence markers, including senescence-associated β-galactosidase activity, DNA damage, lysosomal activity, and oxidative stress indicators such as malondialdehyde. Molecular docking revealed CA’s potential interactions with critical proteins involved in senescence signalling pathways, suggesting mechanisms by which CA may exert its effects. Gene expression and protein analyses corroborated the observed anti-senescent effects, with CA modulating p16, p21, and pRB1 expressions and reducing oxidative stress markers. In conclusion, CA appeared to have senolytic and senomorphic potential in vitro, which could mitigate and reverse SIPS markers in BJ fibroblasts.

## 1. Introduction

Senescence is associated with a gradual decrease in the function of the affected tissues and increased sensitivity to stress. This process involves various cellular types and signalling pathways that influence phenotypic changes [1]. Cellular senescence is thought to be a fundamental mechanism of senescence that contributes to the onset of chronic diseases and age-related dysfunctions [2,3]. While cellular senescence caused by severe intracellular or extracellular stress or damage has been considered to induce irreversible cell cycle arrest, researchers now recognise that senescence is a dynamic, multi-step process [4]. Senescent cells (SCs) increase with age in various mammalian species, including humans [5]. Factors that can cause senescence include repetitive cell division and signals that promote cell growth, telomere shortening, DNA damage and mutations, the accumulation of misfolded proteins, and an increase in reactive oxygen species (ROS) [6,7,8,9]. The free radical theory called the oxidative stress theory, is a well-established mechanism that contributes to cellular senescence [10]. Oxidative stress is a cellular condition that occurs when there is an imbalance between ROS formation and the ability of cells to remove ROS [11]. Oxidising cell metabolism products or known oxidative agents can cause oxidative stress-induced premature senescence (SIPS). Hydrogen peroxide (H_2_O_2_), a widely recognised oxidising agent, triggers oxidative stress and accelerates senescence in several cell types [12,13].

There are some physical features that only SCs possess. They are larger and flatter with rough edges, a giant nucleus, a single, more noticeable nucleolus, and more cytoplasmic vacuoles [14,15]. Additionally, SCs exhibit enhanced activity of senescence-associated β-galactosidase (SA-βgal) [16]. Similar to cancer cells, SCs resist apoptosis [17]. Key anti-apoptotic pathways active in SCs, including B-cell lymphoma 2 (Bcl-2)/Bcl-xL, PI3K/AKT, p53/p21, ephrins, HIF-1α, and HSP-90, illustrate the complex and multifaceted nature of the senescent state [18,19,20]. Senescent cells exhibit excessive production and release of several bioactive peptides, a situation known as the Senescence-Associated Secretory Phenotype (SASP). This phenotype includes many cytokines, chemokines, matrix metalloproteinases, and other proteins [21,22,23]. The SASP has a strong pro-oxidant and pro-inflammatory effect on the microenvironment [15]. Compounds with antioxidant or anti-inflammatory properties may serve as agents to counteract the effects of the SASP [24]. These data indicate that various categories of phytochemicals can regulate the senescence process [25]. Removing SCs is a common approach for dealing with the consequences of senescence [26]. Drug development targeting senescence categorises approaches as senomorphic (inhibiting the SASP to reduce inflammation) and senolytic (selectively eliminating SCs). Senomorphics can prevent the spread of senescence by reducing the expression of SASP-related elements and pathways [27]. The first discovered senomorphics included rapamycin, metformin, resveratrol, and aspirin [28]. Senomorphics affect various biological processes, including SIRT1, mTOR, NF-κB, P38MAPK, glucocorticoid receptors, mitogen-activated protein kinase-activated protein kinase 2 (MK2), MDM2 and IL-1α [29]. Senolytic agents have shown promise for removing SCs, offering therapeutic benefits against senescence and related diseases [30]. Senolytic substances can selectively eliminate SCs, both in vitro and in vivo. They exhibit a favourable therapeutic impact on senescence and age-related ailments [31]. Senolytics target a variety of signalling pathways, including tyrosine kinase receptors (TKRs), growth factor receptors (GFRs), ephrin receptor B1 (EFNB1), SRC kinases, PI3K-AKT, HSP90, members of the Bcl-2 family, and mechanisms involving caspase inhibition and p53 regulation [32]. A novel class of senolytics is being identified using high-throughput library screens and other methods [33].

Chelidonic acid (CA) is found in various plant species, including Chelidonium majus (known as Celandine) and other members of the Papaveraceae family [34]. CA is the γ-pyrone [35], with the pyran ring structure being a critical pharmacophore integrated into various bioactive compounds [36]. These compounds are known for their antitumor, antibiotic, antibacterial, antiallergic lipid-lowering, and immune-modulating effects [37,38]. CA, a highly valued compound in Chinese herbal medicine, CA has been historically recognised for its medicinal benefits [39]. It has several known therapeutic effects, including acting as an anti-inflammatory agent with potential therapeutic applications for allergic disorders [40], as well as having antimicrobial [41] and anticancer effects [42]. Porter et al. reported that CA is a potent inhibitor of glutamate decarboxylase in the rat brain [43]. Additionally, Oh et al. demonstrated that CA controls inflammatory cytokine expression and the activation of caspase-1 in a mouse model of allergic rhinitis [44]. Shin et al. reported that CA inhibited the expression of nuclear factor-κB (NF-κB)-related interleukin-6 (IL-6) [35]. Kim et al. also reported that CA inhibits the symptoms of atopic dermatitis [45]. These findings highlight CA’s comprehensive biological activities that are relevant to our study. The anti-inflammatory and anti-proliferative properties observed in previous studies provide a solid basis for our hypothesis that CA could mitigate senescence-related pathways, thus providing a basis for its use in age-related disease management.

The primary aim of this study was to investigate the therapeutic potential of CA for alleviating and reversing oxidative stress-induced cellular senescence. We chose to focus on CA because it is a γ-pyrone compound with many pharmacological activities and has the potential as both a senomorphic and senolytic agent that can reverse or alleviate markers of cellular senescence. Our research evaluating the effects of CA on SA-βgal activity, indicators of oxidative stress such as MDA, and gene-proteins involved in senescence signalling pathways through expression analysis and molecular docking studies highlights the critical role that CA may play in modulating age-related physiological changes and influencing the pathogenesis of many age-related diseases. These findings provide a basis for in vivo investigations to combat oxidative stress-induced cellular senescence. Additionally, these findings have implications for developing treatments for both the medical and cosmetic industries.

## 2. Materials and Methods

### 2.1. Cells Culture and Treatment

The BJ human cell line (CRL-2522, ATCC) was cultured in T-75 flasks using Dulbecco’s modified eagle medium (11885084-Gibco, Grand Island, NY, USA) supplemented with FBS 10% (10500064-Gibco), MEM Non-Essential Amino Acids Solution 1% (11140050-Gibco) and Penicillin-Streptomycin-10,000 U/mL 1% (15140122-Gibco). Cells were maintained at 37 °C and 5% CO_2_. Morphological changes were analysed by phase contrast microscopy (Zeiss Axio Vert, Oberkochen, Germany); cell viability was determined by trypan blue (15250061-Thermo Fischer Scientific, Grand Island, NY, USA) exclusion test; and cell counts were performed using a thoma lam (Marienfeld, Lauda-Koenigshofen, Germany). The cells were passaged at 70–80% confluency. Cells were harvested using trypsin-EDTA solution (25200056-Gibco). Every three days, the culture medium was washed with PBS (10010015-Gibco), and fresh medium was added to the cells in passages 7 to 12 that were used in the experiments. The SMART™ BCA Protein Assay Kit (21071-Intron biotechnology, Gyeonggi, Republic of Korea) was used for protein quantification in all studies.

### 2.2. Developing a Stress-Induced Premature Senescence (SIPS) Model

In this section, the minimum dose and duration of oxidative stress required to induce premature senescence were determined. A single-stage oxidative stress model was used to induce premature senescence in the BJ cells [46]. Cell doubling time, SA-βgal activity, and sublethal doses of H_2_O_2_ and CA were determined using MTT assays. These steps established a reliable model for studying stress-induced premature senescence and evaluating the effects of CA.

#### 2.2.1. Doubling Time

Approximately 50,000 cells were seeded in a T-25 flask, and cell counting was performed at 24-h intervals in triplicate over ten days. Cells were counted with Thoma lam (Marienfeld). Doubling times were calculated using GraphPad Prism (Version 8.4.0 for Mac, GraphPad Software, San Diego, CA, USA).

#### 2.2.2. Staining Cells for SA-βgal

SA-βgal activity at pH 6 was used to determine the treatments’ model and confirm the doubling time. Cells were seeded at a density of 5 × 10^4^ cells/well, and their density was observed 24 h after seeding. Cell staining was performed on days 3, 6 and 9, according to the results of the doubling time analysis. Senescence β-Galactosidase staining was performed using a commercial kit (ab102534 SA-βgal staining Kit, Abcam, Cambridge, MA, USA). From a randomly selected field of view, 7 images were captured at 4×, 10×, 20× and 40× magnification using a fluorescence microscope (Zeiss Axio Vert). All the procedures were repeated in triplicate.

#### 2.2.3. Determining the Sublethal Dose of H_2_O_2_ as an Oxidative Stress Agent

The sublethal dose range of H_2_O_2_ in the SIPS model was determined using a tetrazolium dye (MTT) assay. This assay measures the mitochondrial dehydrogenase activity. Mitochondrial enzymes in living cells convert the yellow MTT tetrazolium salt to an insoluble purple formazan product, with the amount of formazan correlating with cell viability [47]. BJ fibroblast cells were seeded in 96-well plates at a concentration of 1 × 10^4^ cells per well. Next, plates were incubated at 37 °C and 5% CO_2_. The medium was removed after 24 h, and various concentrations of H_2_O_2_ (100–1000 µM) were added. After 3 h of incubation, the culture media was replaced entirely with a fresh medium containing the tetrazolium dye (T0793-MTT dye, Bio Basic, Markham, ON, Canada) at a 0.5 mg/mL concentration. The plate was incubated at 37 °C and 5% CO_2_ for 4 h. Next, the medium was discarded, and 100 µL of dimethyl sulfoxide (S-002-M- DMSO, Sigma–Aldrich, St. Louis, MO, USA) was added. A multifunctional plate reader was used (Epoch 2, BioTek, Santa Clara, CA, USA) to determine the absorbance at wavelengths of 570 and 690 nm (for background measurements). The experiment was conducted in duplicate. Cell viability was measured by calculating the percentage of formazan absorbance. Cell viability (%) was calculated using the following equation and the corrected data:

*A_contro_*_l_ is the absorbance of the control group at 570–690 nm (without treatment).

*A_treatment_* is the treatment group’s absorbance at 570–690 nm.

$$\mathrm{Cell}\;\mathrm{viability}=100\;\times \;(\;\frac{Atreatment}{Acontrol})$$

Additionally, morphological changes in BJ fibroblast cells after CA treatment were observed using an Axio Zeiss microscope (Zeiss Axio Vert).

#### 2.2.4. Determining the Sublethal Dose of CA

As described above, MTT assay was used to analyse the effects of cytotoxicity on cell proliferation. The cells were seeded in 96-well plates at a density of 1 × 10^4^ cells per well and exposed to CA concentrations ranging from 10 to 4000 µM. Exposure times of 24, 48, and 72 h were considered to evaluate the effect of varying treatment times. Cells in the control group received no treatment; those in the treatment groups were administered the specified concentrations of CA.

### 2.3. Free Radical Measurements

#### 2.3.1. Staining Cells for SA-βgal 

SIPS studies were performed on BJ fibroblast cells cultured at a density of 5 × 10^4^ cells per well. After 24 h of incubation, all experimental groups, except the control group, were treated with 200 μM H_2_O_2_ for 3 h at 37 °C to induce premature senescence. Following the 3-h treatment, the medium in the H_2_O_2_-treated group was replaced with a complete medium. Subsequently, 250 and 500 μM CA concentrations were applied to the respective CA groups for 72 h. The working solutions were prepared in a complete culture medium (319-066CL-DMEM, Wisent, St-Bruno, QC, Canada). Senescence β-Galactosidase staining was conducted using a commercial kit (ab102534 SA-βgal staining Kit, Abcam). For imaging, seven fields of view were randomly selected, and images were obtained at 4×, 10×, and 20× magnifications using a fluorescence microscope (Zeiss Axio Vert). 

#### 2.3.2. Cytotoxicity Testing: AO/DAPI Double Staining

##### 4′,6-Diamino-2-Phenylindole (DAPI) Staining

DAPI (4′,6-diamino-2-phenylindole, 62248, Thermo Fischer Scientific) staining method was used to visualise nuclear morphology and integrity [48]. After SIPS, the culture medium was removed. Next, cells were washed twice with PBS (1X). They were then incubated for 30 min at 37 °C using a fixing reagent (4% paraformaldehyde). Next, the cells were washed twice more with PBS (1X). DAPI (0.5 μg/mL) was added, and fixed cells were incubated for approximately 30 min at 37 °C. The DAPI solution was removed then. Finally, the cells were washed twice with PBS. The binding of the dye to DNA was visualised using fluorescence microscopy (Zeiss Axio Vert). A total of 7 images were obtained (4×, 10×, and 20× magnification) from a randomly selected field of view.

##### Acridine-Orange Staining (AO)

Acridine-orange staining (GT9731, Glentham Life Sciences, Corsham, UK) was used to visualise cellular acidic vacuoles, a reliable marker of autophagy [49]. A total of 1 µg/mL acridine orange solution in PBS was applied for 10 min at 5% CO_2_ and 37 °C. Next, the cells were washed twice with PBS. A total of 7 images (4×, 10× and 20× magnification) were acquired from a randomly selected field of view using a fluorescence microscope equipped with filters for AO (excitation at 488 nm and emission at 590 nm for red fluorescence, Zeiss Axio Vert).

#### 2.3.3. Genotoxicity Testing: Comet Assay

An alkaline comet assay was used to assess DNA damage in BJ cells. The assay was performed according to the manufacturer’s protocol (ab238544 Comet Assay Kit, Abcam). In brief, the cells were gently harvested by scraping the wells (6-well plates). The cells were centrifuged at 1500 rpm using a Hermle centrifuge (Z326K, Wehingen, Germany). 

The resulting cell pellet was remixed with ice-cold PBS to obtain a 1 × 10^5^ cells/mL concentration. The cell solution was mixed with comet agarose and immediately applied to glass slides, pre-coated with a base layer of comet agarose. The slides were suspended in a pre-chilled alkaline solution for 30 min at 4 °C in the dark. Electrophoresis was then performed using a cold alkaline electrophoresis buffer for 40 min. Next, the air-dried slides were incubated with Vista Green DNA dye at room temperature for 15 min and viewed using a fluorescence microscope (Zeiss Axio Vert, 20× magnification). Fifty cells were analysed per sample. The ImageJ OpenComet software v1.3.1 [50] was used to quantify the tail moment and DNA in the tail.

Tail Moment was measured using the following methods:

Tail DNA% = 100 × Tail DNA Intensity/Cell DNA Intensity

(a) Olive Tail Moment = Tail DNA% × Tail Moment Length

(b) Width of Tail Moment = Tail DNA% × Tail Length

#### 2.3.4. H_2_O_2_ Measurements

The quantity of H_2_O_2_ was determined using a Hydrogen Peroxide Test Kit (ab102500, Abcam, Cambridge, MA, USA) according to the manufacturer’s instructions. This test uses horseradish peroxidase, a probe, and H_2_O_2_ to form a coloured product (λmax = 570 nm). Standards were added to each microplate well, and 50 μL of the reaction mix was added to the respective test mixture and the H_2_O_2_ standards. The samples were then incubated at room temperature for 10 min. Absorbance measurements were obtained using a micro-plate reader (Epoch 2, BioTek, Winooski, VT, USA) at 570 nm. The concentration of H_2_O_2_ (in nanomolar concentrations) was determined using a H_2_O_2_ standard curve. The results were expressed in terms of pmol/µL H_2_O_2_. 

#### 2.3.5. Malondialdehyde Levels 

Malondialdehyde is a by-product of lipid peroxidation [51]. A test based on the thiobarbituric acid reagent (TBARS) was used to measure MDA levels [52]. Pre-treated cell lysate (25 µL) was mixed with 175 µL of thiobarbituric acid (T5500-TBA, Sigma–Aldrich, USA) reactive solution containing 50 µL of 0.67% (*w*/*v*) TBA and 125 µL of 20% (*w*/*v*) trichloracetic acid (100807-TCA, Merck, Darmstadt, Germany). The mixture was then heated at 95 °C for 30 min. Next, they were quickly chilled on ice for 10 min and then centrifuged at 3000 rpm for 10 min. The supernatant collected after centrifugation was used for absorbance measurements at 532 nm. The MDA concentration was determined using an MDA calibration curve. The results were expressed as nmol MDA per mg protein in the cells.

### 2.4. Determination of Antioxidant Activity

#### 2.4.1. Superoxide Dismutase Activity

The activity of cellular superoxide dismutase (SOD) was measured according to the principle described by Sun et al. [53]. This principle involves the generation of O_2_^−^ (a superoxide anion) via the reaction of xanthine (X0626, Sigma–Aldrich) with xanthine oxidase (X1875-5UN, Sigma–Aldrich) and the subsequent formation of a coloured compound with nitroblue tetrazolium chloride (ab146262 -NBT, Abcam). The reaction mixture was prepared with a mixture of xanthine (0.3 mmol), NBT (150 μmol/L), sodium carbonate (400 mmol/L, 223530, Sigma–Aldrich), bovine serum albumin (1 g/L, A2153, Sigma–Aldrich), EDTA (0.6 mmol/L, E5134, Sigma–Aldrich), and xanthine oxidase enzyme (0.857 U/mL). Next, 2.5 mL of this mixture was added to 500 µL of cell lysates and then incubated for 20 min at 25 °C. The reaction was stopped by adding 1 mL of cupric chloride (0.8 mmol/L, 751944, Sigma–Aldrich). The absorbance of formazan was quantified at 560 nm against blank (reaction mixture except for the cell lysate). SOD activity was quantified as units per milligram of protein.

#### 2.4.2. Glutathione Peroxidase (GSH-Px) Activity

According to the manufacturer’s instructions, GSH-Px activity was measured with an Elabscience test kit (E-BC-K096-S-Elabscience, Houston, TX, USA). Glutathione activity was calculated by measuring reduced glutathione consumption. H_2_O_2_ and GSH can react without GSH-Px catalysis. Therefore, the part of GSH reduction that occurs by non-enzymatic reaction was removed. GSH can react with dinitrobenzoic acid to form a yellow 5-thio-dinitrobenzoic acid anion. The difference in absorbance value between control and treatment groups indicates GSH-Px activity. The absorbance of the reaction mixture was measured at 412 nm. GSH activity was expressed as U/mg protein. 

### 2.5. Molecular Docking Experiments

The following steps were carried out for molecular docking experiments: to examine the potential senolytic and senomorphic effects of chelidonic acid on premature senescence induced by H_2_O_2_, all cellular senescence-associated proteins were investigated in the literature. The top 12 critical targets in the degree ranking were subjected to molecular docking analysis with one key active ingredient: chelidonic acid. Ligand (Chelidonic acid-PubChem ID:7431) from the PubChem database [54]. Ligand structure converted to pdb format using the Open Babel program (version 2.4.1) [55]. The docking compatible (pdbqt) files of the ligand and the target proteins were created using the AutoDock version 1.5.4 (ADT; Scripps Research Institute, La Jolla, San Diego, CA, USA) [56] with the grid sizes of x, y, z and coordinates used to generate grids that enclose the active sites. All docking results were performed with AutoDock version 1.5.4 (ADT). They were saved in pdbqt format and analysed in Discovery Studio Visualizer version 21.1.0 (Dassault Systèmes, San Diego, CA, USA) [57] and PyMol (DeLano Scientific LLC, Palo Alto, CA, USA) [58] applications.

### 2.6. Gen Expression and Protein Analysis

#### 2.6.1. Real-Time PCR Analysis

##### Total RNA Isolation and First-Strand cDNA Synthesis

Total RNA was extracted using a PureLink™ RNA Mini Kit (Thermo Fisher Scientific) according to the manufacturer’s instructions. The RNA quantity was assessed using a Nanodrop ND-1000 Spectrophotometer (Nanodrop Technologies Inc., Wilmington, DE, USA). The quality of the total RNA was evaluated using an Agilent Bioanalyzer 2100 system (Agilent Technologies, Santa Clara, CA, USA) and the Agilent RNA 6000 Nanochip kits (Agilent Technologies). The instructions provided by Agilent are as follows.

Total RNA (0.3 µg) was reverse transcribed using a RevertAid First Strand cDNA synthesis kit (Thermo Fisher Scientific).

##### Quantitative, Real-Time PCR

The primer pairs selected for amplification were carefully crafted using Primer3web version 4.1.0 “http://primer3.ut.ee/ (accessed on 21 February 2024)” [59]. The details of the primer pairs used for this investigation are provided in Table S1.

Real-time PCR amplification was performed in 96-well plates using the StepOnePlus™ System (Thermo Fisher Scientific). The β-actin gene was used as a housekeeping gene. Each reaction contained 1 μL of 10 ng cDNA template, 5 μM of each primer, and 1× PowerUp™ SYBR™ Green Master Mix (Thermo Fisher Scientific) to yield a final volume of 20 μL. All the reactions were carried out in duplicate for each cDNA sample. A no-template control (NTC) was also included for each gene in every experiment. This experiment was independently repeated twice for all the selected genes in each experiment group’s cell lysate. The PCR running protocol was set at 95 °C for 10 min thermal activation and denaturation, followed by 45 cycles of 95 °C for 10 s and 59 °C for 10 s. A dissociation curve was constructed to determine the specificity of the reactions by increasing the temperature from 65 to 95 °C. The crossing cycle number (Cp) was automatically determined for each reaction using the StepOnePlus™ Real-Time PCR System (Thermo Fisher Scientific).

Melting curve analysis was used to confirm the efficiency and precision of the quantitative PCR (qPCR). Gene expression levels were measured based on the 2^−ΔΔCT^ method to normalise the values [60]. To ensure data reliability, all the experiments were conducted at least twice.

#### 2.6.2. Western Blot Analysis

Western blotting is a pivotal technique for confirming the protein level changes linked to premature senescence induced by oxidative stress [61], as predicted by molecular docking and gene expression analyses. Protein samples were prepared in radioimmunoprecipitation lysis buffer with a protease inhibitor single-use cocktail and phosphatase inhibitor cocktail (78420-Thermo Fisher Scientific). Protein concentrations were measured using the Qubit™ Protein Assay Kits (Q33211-Invitrogen, San Diego, CA, USA). Subsequently, 60 µg of protein was incubated with LDS sample buffer and bold reducing agent at 70 °C for 10 min. Protein samples (60 μg) were loaded onto a Bolt 4–12% Bis-Tris Plus gel (Thermo Fisher Scientific). The gel was then run at 200 V for 20 min. Proteins were transferred onto a nitrocellulose membrane using the iBlot 2 Dry Blotting System (Thermo Fisher Scientific). Primary and secondary antibody reactions were performed using the iBind Flex Western System (Thermo Fisher Scientific, Inc.). The membranes were incubated with iBind Flex Solution (iBind Flex Buffer, iBind Flex Additive and distilled water) for 10 min at room temperature to block nonspecific binding. Primary (p21, p16, pRB1 and β-actin) and secondary (HRP Goat Anti-Rabbit IgG (H + L) and HRP Goat Anti-Mouse IgG (H + L)) antibody reactions were performed at room temperature for 2.5 h, following the manufacturer’s protocol. Protein-antibody complexes were visualised using Pierce ECL Western Blotting Substrate. The immunoreactive protein bands were detected using the GEN-BOX imagER Fx (ERBiyotek, Ankara, Turkey). Antibodies used in this study are listed in Table 1.

### 2.7. Statistical Analysis

The results are presented as mean ± standard error of the mean (SEM) derived from at least three independent experiments. The statistical significance of the results was determined using one-way ANOVA, followed by Tukey’s multiple comparison test. GraphPad Prism 8.4.0 software (GraphPad Software) was used for the analysis. Statistical significance was established at a confidence level of 0.05.

## 3. Results

### 3.1. Developing a Stress-Induced Premature Senescence (SIPS) Model

#### 3.1.1. Doubling Time

**Figure 1:**
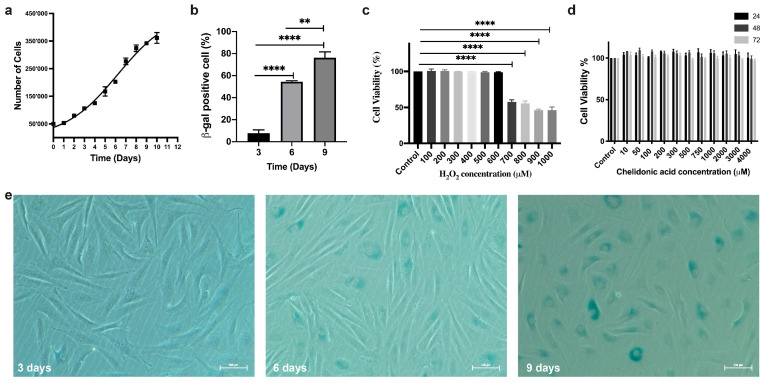
(**a**) Doubling Time of Cell Proliferation. The graph shows an average of doubling times over 10 days. (**b**) Beta-Galactosidase Staining Quantification: Bar graph displaying the percentage of beta-galactosidase-positive cells at days 3, 6, and 9. (**c**) Cell Viability After H_2_O_2_ Exposure. MTT assay results show cell viability after 3 h of exposure to various concentrations of H_2_O_2_. (**d**) CA Impact on Cell Viability. Line graph illustrating the effects of CA on cell viability 24, 48, and 72 h post-treatment, using the MTT assay. (**e**) Beta-Galactosidase Staining Images. Representative images of cells on days 3, 6, and 9 show beta-galactosidase activity indicative of senescence. Data points represent means ± SEM from three independent experiments. Values are analysed using GraphPad Prism software through the one-way ANOVA and Tukey’s Post hoc assay with significant differences noted (** *p* < 0.01, **** *p* < 0.0001).

An exponential growth model was used to examine how the population of BJ fibroblast cells changes over time, exhibiting parameters very close to fibroblast biological growth characteristics. According to our results, doubling time was determined as 3.86 days (Figure 1a). The American Type Culture Collection (ATCC CRL-2522, Manassas, VA, USA) supports our conclusion. 

#### 3.1.2. Staining Cells for SA-βgal

The number of SA-β-gal positive cells (%) was evaluated for days 3, 6, and 9 (Figure 1b). Post hoc analysis was performed with the Tukey test according to a one-way ANOVA result (*p* > 0.05). Tukey’s multiple comparison tests showed significant differences among the groups at different time points. SA-β-Gal positive cells were calculated on day 3 (7.720 ± 2.99%), day 6 (54.43 ± 0.96%) and day 9 (76.24 ± 5.35%). The differences between days 3 and 6 (*p* < 0.0001) and between days 3 and 9 were statistically significant at *p* < 0.0001, as was the difference between days 6 and 9 (*p* = 0.001, *p* < 0.05). These data show a significant increase in the number of SA-β-gal positive cells with time and indicate the onset of cellular senescence, characterised by a gradual decrease in cellular proliferation and irreversible cell cycle arrest, resistance to apoptosis and secretion of pro-inflammatory factors. Furthermore, the apparent time-dependent increase of the senescence phenotype shown in Figure 1e is consistent with doubling times.

#### 3.1.3. Determining the Sublethal Dose of H_2_O_2_ as an Oxidative Stress Agent

To determine the sublethal dose of H_2_O_2_ as an oxidative stress agent, a concentration range of 50–1000 µM was evaluated based on MTT test results. Post hoc analysis was performed with the Tukey test according to a one-way ANOVA result (*p* > 0.05). Tukey’s test results showed that 50–600 µM H_2_O_2_ had no significant effect on cell viability than the control group, indicating that suggests that at these concentrations, H_2_O_2_ effectively may induce oxidative stress, leading to cellular dysfunction and possible initiation of senescence pathways. However, the 700–1000 µM H_2_O_2_ concentration range exhibited a significant antiproliferative effect, as indicated by reduced cell viability, with the difference between these concentrations and the control group being statistically significant at the *p* < 0.0001 level (Figure 1c). Given the need to induce oxidative stress without causing cell death, 200 µM H_2_O_2_ was chosen as the treatment dose, as this would confound the results by introducing necrosis or apoptosis instead of true senescence. Data from the literature and preliminary study results support this concentration, as it provides a balance where oxidative stress is sufficient to induce senescence while maintaining cell viability [62]. The choice of 200 µM H_2_O_2_ allows for the study of stress-induced premature senescence (SIPS) in a controlled manner, facilitating the exploration of chelidonic acid’s potential to modulate senescence pathways without overwhelming the cells with cytotoxic effects.

#### 3.1.4. Determining the Sublethal Dose of CA

The sublethal dose range of CA on BJ cell viability was evaluated according to the mtt test results in the 10–4000 µM dose range. Since the data were equal and the variances were homogeneous according to the one-way ANOVA result, the Tukey Test performed Post hoc analysis (*p* > 0.05). The analysis showed no statistically significant difference between the control and any CA dose range applied for 24, 48 and 72 h (*p* > 0.05). MTT and Tukey test results revealed that the 10–1000 µM CA dose range had no anti-proliferative effect on BJ cells (Figure 1d) [44]. This result indicates that it is suitable for investigating its effect on cellular senescence. Based on the balance between the potential effect range of CA and cell viability, 250 and 500 µM CA concentrations were strategically selected for the subsequent experiments. These concentrations were chosen to investigate the effects on senescence and related pathways without cytotoxic effects, and the CA treatment time was set at 72 h to evaluate the long-term effects of CA on cellular senescence in vitro and treatment time allowed the evaluation of the potential of CA to regulate cellular senescence over a long treatment period.

### 3.2. Free Radical Measurements

#### 3.2.1. Staining Cells for SA-βgal

**Figure 2:**
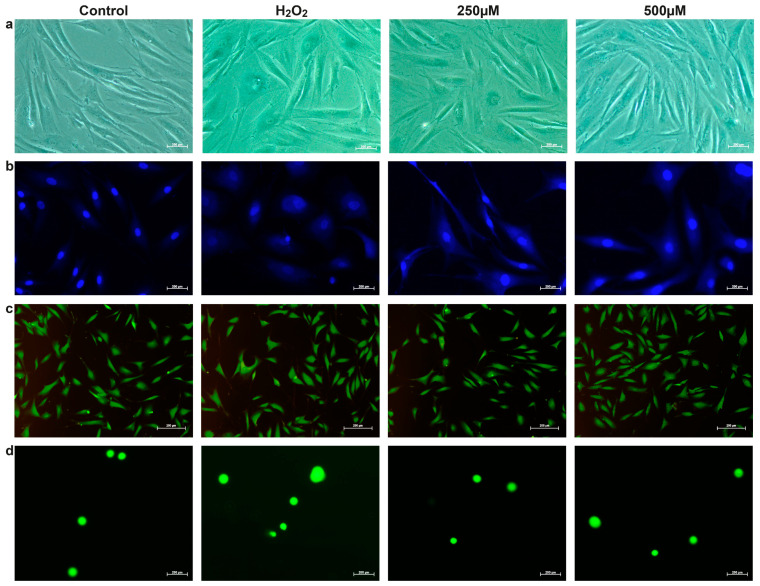
(**a**) Representative Beta-Galactosidase staining microscopy images illustrating varying levels of senescence. (**b**) Representative fluorescence DAPI staining images assessing changes in nuclear morphology and chromatin organisation. (**c**) Representative Acridine orange staining images are highlighted as markers of DNA damage and lysosomal activity. (**d**) Representative Comet assay images showing DNA damage levels via nuclear morphology and DNA migration patterns. These images highlight the effects of each treatment on cellular senescence, nuclear morphology, and DNA integrity.

**Figure 3:**
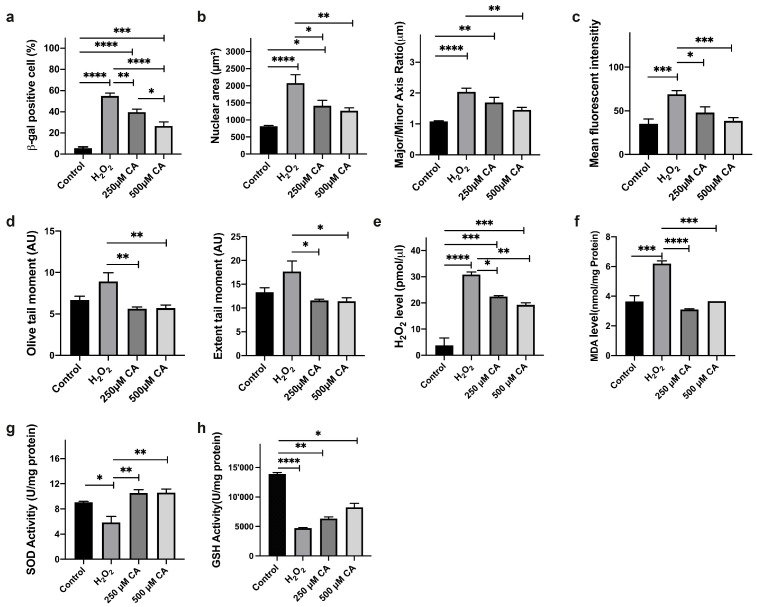
(**a**) Beta-Galactosidase Staining Quantification, bar graph showing the percentage of beta-galactosidase-positive cells. (**b**) Quantitative analysis of DAPI staining images of the nuclear area. The nuclear area and Major-Minor Axis ratio of the CA-treatment and control groups show similar trends, with the CA-treatment and control groups showing smaller nuclear size than the H_2_O_2_-treatment group. (**c**) Quantitative analysis of Red Fluorescence in Acridine Orange-Stained Cells for lysosomal activity. This figure presents the statistical analysis of red fluorescence intensity in acridine orange (AO)-stained cells after SIPS, CA treatment. (**d**) Quantitative analysis of comet assay images, including tail DNA percentages and comet tail lengths. Each image contains multiple comets, and the study provides detailed information on DNA damage. (**e**) H_2_O_2_ Measurement: Graph showing intracellular hydrogen peroxide levels, illustrating oxidative stress modulation by each treatment. (**f**) MDA Levels A bar graph of MDA levels indicates lipid peroxidation under different treatments. (**g**) SOD Levels, line graph displaying SOD activity, reflecting antioxidant defence mechanisms. (**h**) GSH Levels, plot of GSH concentrations, assessing antioxidant capacity in response to treatments (*n* = 3). The data are means ± SEM from three independent experiments. Values are analysed using GraphPad Prism software through the one-way ANOVA and Tukey’s Post hoc assay with significant differences noted (* *p* < 0.05, ** *p* < 0.01, *** *p* < 0.001, **** *p* < 0.0001). This figure highlights how treatments affect cellular redox balance and lysosomal and antioxidant activities.

The CA effect on increased SA-βgal activity was investigated. According to the one-way ANOVA result, the Tukey Test performed Post hoc analysis (*p* > 0.05). The analysis revealed that the difference between the control (5.61 ± 1.25) and the H_2_O_2_-treated groups (54.92 ± 2.64) was statistically significant (*p* < 0.0001), confirming that H_2_O_2_ effectively increases senescence marker expression (Figure 2a and Figure 3a). Additionally, significant differences were observed between the control and the 250 µM CA (39.68 ± 2.84) and 500 µM CA (26.60 ± 3.84) treatment groups, with *p*-values of <0.0001 and 0.0004, respectively. Moreover, there was a statistically significant reduction in SA-β-Gal-positive cells when comparing the H_2_O_2_ (54.92 ± 2.64) to the 250 µM CA (39.68 ± 2.84) (*p* = 0.0070, *p* < 0.05) and the 500 µM CA (26.60 ± 3.84) groups (*p* < 0.0001). This reduction indicates that CA treatment can decrease the senescence marker SA-β-Gal activity dose-dependently. In addition, the difference between 250 µM CA (39.68 ± 2.84) and 500 µM CA (26.60 ± 3.84) groups was statistically significant (*p* = 0.0212, *p* < 0.05). A total of 250 µM CA treatment reduced the percentage of SA-β-Gal positive cells by 27.76 ± 2.84% compared to the H_2_O_2_ group. In comparison, 500 µM CA treatment reduced it by 51.57 ± 3.84% (Figure 3a). These results suggest that CA has a potential senomorphic effect against the SASP marker by regulating the pathways that reduce SA-βgal activity.

#### 3.2.2. DAPI-Acridine Orange (AO) Staining

The DAPI staining technique was used to investigate the potential attenuating effects of CA on nuclear senescence phenotypes. DAPI-stained images were analysed using FIJI/ImageJ v 2.14.0/1.54f, and nuclear area-major and minor axis ratios were determined (Figure 3b). The Post hoc Tukey Test was performed according to one-way ANOVA results (*p* > 0.05). The H_2_O_2_-treated group exhibited larger and more irregular nuclear structures than the control and CA-treated groups (Figure 2b). A significant difference in nuclear area and axis ratios was found between control and H_2_O_2_-treated groups at *p* < 0.0001 significance level, indicating significant nuclear changes due to senescence. Similarly, a significant difference was observed between control and 250 µM CA-treated groups with *p*-values of 0.0363 and 0.0013, respectively, emphasising the effect of CA in ameliorating these senescence-related changes. However, the difference between the control and 500 µM CA-treated groups was insignificant (*p* = 0.1623 and *p* = 0.0944), suggesting that higher CA concentration may stabilise nuclear morphology and showed similar results to the control group.

A significant difference in the nuclear area was noted between the H_2_O_2_ and 250 µM CA treated groups (*p* = 0.0163), but no significant difference in axis ratios was observed (*p* = 0.1382, *p* > 0.05). In contrast, H_2_O_2_ and 500 µM CA-treated groups showed statistically significant differences in the two parameters (*p* = 0.0023), emphasising the potential of CA to reverse senescence-related nuclear changes. No significant difference was observed between the 250 µM and 500 µM groups treated with CA (*p* = 0.9091 and *p* = 0.4182, *p* > 0.05), suggesting that both concentrations effectively reduced senescence markers to a similar extent.

Nuclear expansion is a known feature of cellular senescence. It is associated with the cell’s inability to divide and also reflects the accumulation of DNA damage and chromatin reorganisation. The reduction in nuclear size and the repair of nuclear morphology upon CA treatment suggest that CA counteracts these senescence-related nuclear changes, potentially reviving nuclear function and cellular homeostasis.

On the other hand, acridine orange staining (AO) was used to investigate the CA effects on DNA damage and lysosomal activity, which are important cellular senescence indicators. AO-stained images were analysed using FIJI/ImageJ (Figure 3c). According to one-way ANOVA results (*p* > 0.05), the Post hoc Tukey test was performed. The red fluorescence intensity, a marker of DNA damage and lysosomal activity, increased significantly (*p* = 0.0001) in the H_2_O_2_-treated group than the control group, indicating the induction of oxidative stress and associated senescence phenotype. There was no significant difference in red fluorescence intensity between the control and 250 µM CA-treated groups (*p* = 0.2851) and between the 500 µM CA-treated groups (*p* = 0.9675), indicating that CA treatment showed similar results to the control group.

On the contrary, red fluorescence intensity was significantly reduced in the 250 µM CA-treated group than in the H_2_O_2_-treated group (*p* = 0.0262). At the same time, a more significant reduction was observed in the 500 µM CA-treated group (*p* = 0.0006). No significant difference was observed between the 250 µM and 500 µM CA-treated groups (*p* = 0.5384), indicating that both concentrations effectively reduced the H_2_O_2_-induced increase in lysosomal activity and DNA damage.

Quantitative analysis of red fluorescence in AO-stained cells reveals significant differences in RNA and single-stranded DNA content between treated and control groups. This result provides a reliable measure of cellular responses to treatments, including apoptosis and autophagy. The decrease in red fluorescence in CA-treated cells indicates a decrease in the senescence-related SASP marker lysosomal activity. Lysosomal activity is known to increase in senescent cells as part of the degradation of cellular components, and excessive activity may contribute to the pro-inflammatory environment characteristic of senescence. The observed decrease in lysosomal activity after CA treatment suggests that CA may help alleviate SASP and restore a more balanced cellular environment, thus counteracting the senescence phenotype in BJ fibroblasts (Figure 2c).

#### 3.2.3. Comet Assay

The potential role of CA in SIPS-associated DNA damage repair was investigated. The findings were presented using fluorescently stained comet images and included two critical parameters: the olive tail moment and the extent tail moment. Post hoc analysis was performed with the Tukey Test according to a one-way ANOVA result (*p* > 0.05). For the statistical analysis results, *p* values for olive tail moment and extent tail moment are given respectively (Figure 3d). The difference between the control and H_2_O_2_-treated groups was insignificant (*p* = 0.0756, *p* = 0.1069). The comparison between the control and 250 µM CA-treated cells did not show a statistically significant difference (*p* = 0.6337, *p* = 0.7654). Similarly, the difference between the control and 500 µM CA-treated cells was insignificant (*p* = 0.6911, *p* = 0.7119). The difference in the olive tail moment between H_2_O_2_-treated and 250 µM CA-treated groups was statistically significant (*p* = 0.0058, *p* = 0.0143). There was also a statistically significant difference between the H_2_O_2_-treated and 500 µM CA-treated groups (*p* = 0.0072, *p* = 0.0116). The comparison between 250 µM and 500 µM CA-treated groups did not show a statistically significant difference (*p* = 0.9997).

The statistical analysis reveals significant differences in DNA damage between the H_2_O_2_-treated and CA-treated groups. Both 250 µM and 500 µM CA concentrations significantly mitigate DNA damage. However, there is no significant difference in the potential effects between the two CA concentrations (Figure 2d). However, the CA-treated group exhibited minimal dose-dependent accumulation of DNA damage than the H_2_O_2_ group (Figure 2d). This finding suggests that CA may be necessary to repair DNA damage caused by SIPS.

#### 3.2.4. H_2_O_2_ Measurements 

The effects of CA on ROS production and antioxidant activity in H_2_O_2_-treated cells were evaluated. Post hoc analysis was performed with the Tukey Test according to a one-way ANOVA result (*p* > 0.05). As a result of the analysis, the difference between the control (3.78 ± 2.78) and the H_2_O_2_ groups (30.82 ± 0.98) was statistically significant (*p* < 0.0001). A substantial increase in intracellular H_2_O_2_ levels was noted in H_2_O_2_ groups than the control group, suggesting that oxidative stress was effectively induced. The differences between the control and 250 μM CA (22.44 ± 0.33) and 500 μM CA groups (19.26 ± 0.80) were also found statistically significant at 0.0001 and 0.0005 significance levels, respectively. The difference between H_2_O_2_ (30.82 ± 0.98) and 250 μM CA groups (22.44 ± 0.33) was statistically significant (*p* = 0.0205), the difference between H_2_O_2_ and 500 μM CA groups (19.26 ± 0.80) was statistically significant (*p* = 0.0032). Our findings revealed that CA treatment resulted in a dose-dependent decrease in the increased H_2_O_2_ level (Figure 3e). The CA’s ability to reduce ROS levels in a dose-dependent manner highlights its potential as a therapeutic agent in mitigating oxidative stress-related damage, which is a key factor in cellular senescence. In comparison, the difference between 250 μM CA (22.44 ± 0.33) and 500 μM CA (19.26 ± 0.80) was not statistically significant (*p* = 0.5025, *p* > 0.05). By lowering intracellular ROS, CA may help preserve cellular integrity and function, delaying the onset of senescence-related dysfunctions. These results underscore the therapeutic promise of CA in managing conditions associated with oxidative stress and senescence. Results were calculated using a calibration curve and presented in pmol/µL.

#### 3.2.5. MDA Levels

For the MDA level analyses, TBARS levels were investigated as a marker of lipid peroxidation and an indicator of oxidative stress and cellular damage. Post hoc analysis was performed with the Tukey test according to a one-way ANOVA result (*p* > 0.05). As a result of the analysis, the difference between the control (3.64 ± 0.39) and the H_2_O_2_-treated groups (6.19 ± 0.18) was statistically significant (*p* = 0.0002). H_2_O_2_ treatment resulted in an increase in the intracellular level of TBARS compared to the control. There was no statistically significant difference between the control and 250 μM CA (3.10 ± 0.04) and 500 μM CA (3.67 ± 0.00) treated groups at 0.3572 and 0.9998 significance levels, respectively (*p* > 0.05). A statistically significant difference (*p* < 0.0001) was found between H_2_O_2_ (6.19 ± 0.18) and 250 μM CA-treated groups (3.10 ± 0.04). A statistically significant difference (*p* = 0.0002) was also found between H_2_O_2_ (6.19 ± 0.18) and 500 μM CA-treated groups (3.67 ± 0.00). Subsequent CA treatment significantly mitigated the TBARS level. The reduction in TBARS levels following CA treatment suggests that CA effectively counteracts lipid peroxidation, a critical process in developing oxidative stress-related cellular damage, including senescence (Figure 3f). The difference between 250 μM CA (3.10 ± 0.04) and 500 μM CA-treated groups (3.67 ± 0.00) was not statistically significant (*p* = 0.3238, *p* > 0.05). By protecting cellular lipids from peroxidative damage, CA may help maintain cellular integrity and function, potentially delaying the onset of senescence-related dysfunctions. These findings highlight the potential of CA as a therapeutic agent in managing senescence and its associated cellular deterioration.

### 3.3. Antioxidant Activity 

The chelidonic acid treatment effect on the antioxidant system was investigated by measuring SOD activity. The Post hoc analysis was performed using the Tukey test according to a one-way ANOVA result (*p* > 0.05). The difference between the control (9.04 ± 0.16) and the H_2_O_2_-treated groups (5.86 ± 0.92) was statistically significant (*p* = 0.0263, *p* < 0.05). The H_2_O_2_-treated group exhibited a decrease in SOD activity than the control group, as this result confirms. The statistical difference between the control and 250 μM (10.53 ± 0.53) and 500 μM CA-treated (10.58 ± 0.57) groups was not significant at significance levels of 0.3765 and 0.3521, respectively (*p* > 0.05), suggesting that the CA-treated groups showed similar results to the control group. The difference between the H_2_O_2_-treated (5.86 ± 0.92) and the 250 μM CA-treated groups (10.53 ± 0.53) was statistically significant (*p* = 0.0029, *p* < 0.05). The difference between the H_2_O_2_-treated (5.86 ± 0.92) and 500 μM CA-treated groups (10.58 ± 0.57) was also statistically significant (*p* = 0.0028, *p* < 0.05). This increase in SOD activity following CA treatment suggests that CA improves the cell’s antioxidant defence mechanisms. As a result of reducing oxidative stress and related cellular damage. The difference between the 250 μM CA-treated (10.53 ± 0.53) and the 500 μM CA-treated (10.58 ± 0.57) groups was not statistically significant (*p* = 0.9999, *p* > 0.05) (Figure 3g). These findings highlight the potential of CA as a therapeutic agent for maintaining cellular health and preventing the onset of senescence-related dysfunctions.

In addition, the CA treatment effect on GSH-Px activity was also assessed. The Post hoc Tukey test was conducted based on the one-way ANOVA test result (*p* > 0.05). This analysis revealed a statistically significant reduction in GSH-Px activity in the H_2_O_2_-treated group (4712 ± 104) than the control group (13,912 ± 217) (*p* < 0.0001). This result indicates a decrease in GSH-Px activity in the H_2_O_2_-treated group compared to the control group, reflecting a compromised antioxidant defence system. Additionally, a significant difference was observed between the control group and the 250 μM CA (6334 ± 299) and 500 μM CA (8241 ± 681) groups, with *p*-values of 0.0060 and 0.0410, respectively (*p* < 0.05). However, the difference between the H_2_O_2_-treated group and the 250 μM and 500 μM CA-treated groups was not statistically significant (*p* = 0.0806, *p* = 0.0878, *p* > 0.05). However, CA treatment restored GSH-Px activity was observed. Similarly, no significant difference was found between the 250 μM and 500 μM CA-treated groups (*p* = 0.0946, *p* > 0.05). The recovery of GSH-Px activity was dose-dependent in the CA-treated groups (Figure 3h).

### 3.4. Molecular Docking

Molecular docking analysis was performed to investigate the potential senolytic and senomorphic effects of CA on SIPS. The CA interaction with 12 essential proteins was analysed with AutoDock. The PDB code of selected proteins, binding energies and docking parameters are detailed in Table 2. Figure S1 shows the binding patterns between proteins and CA.

The analysis targeted essential proteins known for their roles in stress response and senescence (e.g., pRB1, p21, p16, and p53). In particular, CA demonstrated strong interactions with p21, p16, and p53 proteins, with docking scores of −12.16, −11.99 and 10.13 kcal/mol, respectively. These interactions, which included various forces such as Van der Waals forces, conventional hydrogen bonds, Pi-Alkyl, Pi-Anion, Pi-Cation, and Pi-Sigma, suggest that CA has a high potential to modulate essential senescence-related pathways and help maintain cellular homeostasis.

The molecular docking results provide significant insights into how CA could modulate critical pathways involved in cellular senescence and stress responses. The results suggest that CA has therapeutic potential in managing cellular senescence-related conditions and related disorders and position it as an essential candidate for further research.

### 3.5. Gene Expression and Protein Analysis Results

#### 3.5.1. RT PCR Results

##### Evaluation of Total RNA Isolation Results

The purity of all RNA samples was assessed using absorbance ratios of OD260/280 and OD260/230. Only RNA samples with an OD260/280 ratio (protein contamination) between 1.8 and 2.0 and OD260/230 (organic pollutant) higher than 2.0 were used for further analysis. The purity and integrity of the RNA samples were assessed, and BJ fibroblast cells were derived using a Bioanalyzer 2100 (Agilent Technologies). RNA quality was assessed using RNA Integrity Number (RIN) [75]. The RIN values for all groups were between 8.9 and 9.2.

##### Selection of Candidate Reference Genes and Evaluation of Amplification Specificity, PCR Efficiency, and Gene Expression Levels

To identify the SIPS markers, 11 genes were selected as potential target genes based on a combination of senescence-related data and molecular docking analysis results. All the gene primer sequences are shown in Table S1. The specificity of the RT-qPCR primers was verified by melting curve analyses to provide specific quantitative analyses. 

The expression levels of the 12 selected potential target and reference genes were calculated using Ct values. The Ct values for these reference genes ranged from 15.3 to 34.7. β-actin was identified as the gene with the highest expression among the candidates; it exhibited the lowest Ct value. 

This analysis summarised the statistical significance of all gene expressions under CA treatment after SIPS, respectively, followed by a one-way ANOVA and a post hoc Tukey’s multiple comparison test. 

This analysis revealed the statistical data on p21 gene expression. There was no statistically significant difference in the p21 expression between the control and H_2_O_2_-treated groups (*p* = 0.5447). A comparison between the control and 250 µM CA-treated groups did not show a statistically significant difference (*p* = 0.4533). Similarly, no significant difference was observed between the control and 500 µM CA-treated groups (*p* = 0.1989). The difference between the H_2_O_2_-treated and 250 µM CA-treated groups was insignificant (*p* = 0.0733). However, a statistically significant difference was observed between the H_2_O_2_-treated and 500 µM CA-treated groups (*p* = 0.0290), suggesting that CA at higher concentrations may partially prevent oxidative stress-induced up-regulation of p21, an important regulator of the senescence pathway. While the comparison between 250 µM and 500 µM CA-treated cells showed no statistically significant difference (*p* = 0.9085), this finding indicated a dose-dependent effect of CA in modulating senescence markers. These findings imply that CA may have a protective role against oxidative stress-induced premature senescence, particularly through modulation of the p53-p21 pathway, and highlight its potential as a therapeutic agent for the management of cellular senescence and related pathologies.

This analysis revealed statistical data on p16 gene expression. There was no statistically significant difference in p16 expression between the control and H_2_O_2_-treated groups (*p* = 0.2838). The comparison between the control and 250 µM CA-treated groups showed no statistically significant difference (*p* = 0.8562). The data suggest subtle but potentially biologically relevant shifts in expression. There was a statistically significant difference between the control and 500 µM CA-treated groups (*p* = 0.0028). The difference between H_2_O_2_-treated and 250 µM CA-treated groups was insignificant (*p* = 0.6707). There was a statistically significant difference between the H_2_O_2_-treated and 500 µM CA-treated groups (*p* = 0.0004). p16 is a critical cell cycle regulator, acting as a tumour suppressor by inhibiting cyclin-dependent kinases and inducing cell cycle arrest. This down-regulation suggests that higher concentrations of CA may affect the p16 pathway, potentially attenuating the cell’s tendency to enter the senescent state in response to oxidative stress. The comparison between 250 µM and 500 µM CA-treated groups showed a statistically significant difference (*p* = 0.0012), underscoring the CA dose-dependent effect on this crucial senescence marker.

This analysis revealed statistical data on pRB1 gene expression. There was a statistically significant difference in pRB1 expression between the control and H_2_O_2_-treated groups (*p* = 0.0032), suggesting that H_2_O_2_-induced oxidative stress triggers the upregulation of pRB1, which is consistent with its role in enforcing cell cycle arrest during senescence. The comparison between the control and the 250 µM CA-treated groups showed no statistically significant difference (*p* = 0.9885). The difference between the control and the 500 µM CA-treated groups was also insignificant (*p* = 0.5586). The difference in the pRB1 expression between the H_2_O_2_-treated and 250 µM CA-treated groups was statistically significant (*p* = 0.0062), indicating that this concentration of CA might suppress pRB upregulation. It was observed that pRB1 expression was significantly lower in the 500 µM CA-treated group than the H_2_O_2_-treated group (*p* = 0.0002). These data suggest that CA has a dose-dependent effect against oxidative stress. There was no statistically significant difference in pRB1 expression between the 250 µM and 500 µM CA-treated groups (*p* = 0.3819), indicating that both CA doses can prevent the stress-induced increase in pRB1. However, the effect may reach a limit at higher concentrations. These findings highlight the potential role of CA in modulating the pRB1 pathway, which is crucial for controlling cell cycle progression and senescence. By downregulating pRB1 expression in response to oxidative stress, CA may help prevent permanent cell cycle arrest and offer potential therapeutic benefits in conditions where senescence contributes to disease.

This analysis revealed statistical data on p53 gene expression. There was a statistically significant difference in p53 expression between the control and the H_2_O_2_-treated groups (*p* = 0.0007), indicating that oxidative stress upregulates p53, thereby triggering cellular stress responses. The p53 is well known as a tumour suppressor that governs crucial processes. This increase suggests that H_2_O_2_ activates p53 to initiate a stress response in cells, which could lead to cellular senescence or cell death. The comparison between the control and 250 µM CA-treated groups showed no statistically significant difference (*p* = 0.8179). The difference between the control and 500 µM CA-treated groups was insignificant (*p* = 0.9604). However, a significant reduction in p53 expression was observed when comparing the H_2_O_2_-treated group with the 250 µM CA-treated group (*p* = 0.0029), indicating that CA at this dose may alleviate the p53 activation H_2_O_2_ induced. There was a statistically significant difference in expression between the H_2_O_2_-treated and the 500 µM CA-treated groups (*p* = 0.0003). This suggests that CA may more effectively modulate p53 pathways at higher concentrations. The comparison between the 250 µM and the 500 µM CA-treated groups showed no statistically significant difference in p53 expression (*p* = 0.5442), indicating that both doses affect p53 expression similarly, with the effect potentially reaching a limit at higher concentrations. Given that p53 is activated in response to cellular stress and engages in crosstalk with various other cellular pathways, CA’s ability to suppress p53 upregulation highlights its potential as a therapeutic agent. This suggests that CA could be considered a protective agent in pathological conditions in which p53 has a dominant role.

This analysis revealed statistical data on SIRT1 gene expression. There was no statistically significant difference in SIRT1 expression between the control and the H_2_O_2_-treated groups (*p* = 0.9964), indicating that oxidative stress did not significantly suppress SIRT1 levels. There was also no statistically significant difference between the control and 250 µM CA-treated groups (*p* = 0.2556). However, a statistically significant difference was found between the control and 500 µM CA-treated groups (*p* = 0.0052). The difference in SIRT1 expression between the H_2_O_2_-treated and 250 µM CA-treated groups was not statistically significant (*p* = 0.1863), indicating that low doses of CA may not be sufficient to counteract the effects of oxidative stress through SIRT1. There was a statistically significant difference in expression between the H_2_O_2_-treated and 500 µM CA-treated groups (*p* = 0.0036). This finding suggests that CA at higher concentrations may effectively upregulate SIRT1 and potentially protect against oxidative damage by activating associated antioxidant pathways. Comparison between the 250 µM and 500 µM CA-treated groups showed no statistically significant difference in SIRT1 expression (*p* = 0.1494), suggesting that while SIRT1 expression is increased by CA treatment, the effect may not be dose-dependent within this range. These results emphasise the potential role of CA in modulating SIRT1 activity, especially at high concentrations. Modulation of SIRT1 is particularly important given its critical role in maintaining cellular health and combating the deleterious effects of reactive oxygen species.

This analysis revealed statistical data on PARP1 gene expression. There was no statistically significant difference in PARP1 expression between the control and H_2_O_2_-treated groups (*p* = 0.3161), suggesting that H_2_O_2_-induced oxidative stress may not be sufficient to alter PARP1 levels significantly. The comparison between the control and 250 µM CA-treated groups showed no statistically significant difference (*p* = 0.9549). The difference between the control and 500 µM CA-treated groups was also insignificant (*p* = 0.8773). On the other hand, the difference in PARP1 expression between H_2_O_2_-treated and 250 µM CA-treated groups was not statistically significant (*p* = 0.5601). There was also no statistically significant difference in expression between the H_2_O_2_-treated and 500 µM CA-treated groups (*p* = 0.6908). The between the 250 µM and 500 µM CA-treated groups showed no statistically significant difference in PARP1 expression (*p* = 0.9951). These findings suggest that PARP1, an important part of the DNA damage response and repair pathway, remained unaffected by oxidative stress and CA treatment. The lack of significant changes in PARP1 expression suggests that while CA might exert effects through other molecular mechanisms or pathways, it does not seem to modulate the PARP1 pathway directly. This finding could indicate that the protective or therapeutic potential of CA observed in other contexts might involve pathways other than those directly related to PARP1-mediated DNA repair.

This analysis revealed statistical data on APEX1 gene expression. There was no statistically significant difference in APEX1 expression between the control and H_2_O_2_-treated groups (*p* = 0.7443), suggesting that oxidative stress did not significantly replace APEX1 levels. The comparison between the control and 250 µM CA-treated groups showed no statistically significant difference (*p* = 0.9879). However, there was a statistically significant difference between the control and 500 µM CA-treated groups (*p* = 0.0268), suggesting that at higher concentrations, CA may enhance the activity of DNA repair pathways, possibly offering protection against genotoxic stress. The difference in APEX1 expression between H_2_O_2_-treated and 250 µM CA-treated groups was not statistically significant (*p* = 0.5669), indicating that the lower dose of CA might not be sufficient to counteract the oxidative stress effects at the level of APEX1 regulation. There was no statistically significant difference in expression between the H_2_O_2_-treated and 500 µM CA-treated groups (*p* = 0.1118). This finding suggests that H_2_O_2_-induced oxidative stress did not significantly change APEX1 levels. Nevertheless, the higher CA dose could upregulate APEX1, enhancing the cell’s ability to repair oxidative DNA damage. The comparison between 250 µM and 500 µM CA-treated groups showed a statistically significant difference in APEX1 expression (*p* = 0.0173). These results underline the potential role of chelidonic acid in modulating APEX1 expression. Modulation of APEX1 is important, given its key role in the maintenance of genomic integrity, suggesting that it may provide therapeutic benefits in situations where the DNA repair capacity of CA is compromised.

This analysis revealed statistical data on NRF2 gene expression, which plays a pivotal role in regulating the antioxidant response and cellular defence mechanisms against oxidative stress. There was no statistical difference in NRF2 expression between the control and H_2_O_2_-treated groups (*p* = 0.2317), suggesting that H_2_O_2_-induced oxidative stress might not be sufficient to trigger the downregulation of NRF2 under these conditions. However, the control and 250 µM CA-treated groups showed a statistically significant difference (*p* = 0.0021), indicating that at even lower concentrations, the CA can enhance NRF2 activity. There was a statistically significant difference between the control and 500 µM CA-treated groups (*p* < 0.0001), suggesting CA dose-dependent activation of NRF2. The difference between the H_2_O_2_-treated and 250 µM CA-treated groups was statistically significant (*p* = 0.0300), indicating that the CA can efficiently upregulate NRF2 in the presence of oxidative stress. There was also a statistically significant difference in expression between H_2_O_2_-treated and 500 µM CA-treated groups (*p* = 0.0006). This finding highlights the potential of CA to enhance the cell’s antioxidant defence response. The between 250 µM and 500 µM CA-treated groups showed a statistically significant difference in NRF2 expression (*p* = 0.0434), emphasising the dose-dependent nature of CA’s effects on NRF2 activation. These findings underline the potential for CA to enhance NRF2-mediated antioxidant responses. Given the central role of NRF2 in protecting cells from oxidative damage, the ability of CA to upregulate this pathway suggests that it may provide significant protective benefits against oxidative stress and is a promising candidate for therapeutic strategies aimed at enhancing cellular robustness.

This analysis revealed statistical data on AMPK gene expression, an important regulator of cellular energy homeostasis and metabolic stress responses. There was a statistically significant difference in AMPK expression between control and H_2_O_2_-treated groups (*p* = 0.0010), suggesting that AMPK activity was suppressed and could indicate a reduction in the cell’s capacity to manage energy levels. The difference between the control and 250 µM CA-treated groups was statistically significant (*p* = 0.0002). The difference between the control and 500 µM CA-treated groups was not significant (*p* = 0.9994), suggesting that the 500 µM CA-treated group showed a similar level of AMPK expression to the control group (*p* = 0.9994). The difference in AMPK expression between H_2_O_2_-treated and 250 µM CA-treated groups was not statistically significant (*p* = 0.5300). However, an important difference was observed in the expression between the H_2_O_2_-treated and 500 µM CA-treated groups (*p* = 0.0011), suggesting that higher CA doses may modulate AMPK expression and improve cell adaptation against oxidative stress. The comparison between the 250 µM and 500 µM CA-treated groups showed a statistically significant difference in AMPK expression (*p* = 0.0003), suggesting that CA has a dose-dependent effect and that CA higher concentrations can strongly affect AMPK activity. These findings suggest that CA may significantly affect AMPK pathways that have an important role in cellular energy balance and managing metabolic stress. The CA’s capacity to modulate AMPK may be important for developing and increasing the resilience of cells to stress and improving metabolic health.

This analysis revealed statistical data on BAX gene expression, an essential pro-apoptotic factor regulating cellular death pathways. A statistically significant difference in BAX expression was observed between the control and H_2_O_2_-treated groups (*p* = 0.0014). This result supports a significant decrease in BAX expression in the H_2_O_2_-treated group compared to the control group (*p* = 0.0014). Also, it indicates that cells exposed to senescence develop resistance to apoptosis. The comparison between the control and 250 µM CA-treated groups also showed a statistically significant difference (*p* = 0.0009). However, the difference between the control and 500 µM CA-treated groups was insignificant (*p* = 0.5856). This finding suggests that higher doses of CA may maintain cellular homeostasis by upregulating BAX in senescent cells. The difference in BAX expression between the H_2_O_2_-treated and 250 µM CA-treated groups was not statistically significant (*p* = 0.9804), suggesting that low doses of CA do not alleviate the oxidative stress-induced reduction of BAX. There was a statistically significant difference in BAX expression between the H_2_O_2_-treated and 500 µM CA-treated groups (*p* = 0.0062). Comparison between the groups treated with 250 µM and 500 µM CA showed a statistically significant difference in BAX expression (*p* = 0.0039), suggesting a dose-dependent effect of CA. These findings suggest that chelidonic acid may decrease the capacity of cells to develop resistance to apoptosis by modulating BAX expression. The fact that the 500 µM CA group showed results close to the control group emphasises the protective role of CA in cellular balance and suggests that it should be evaluated as a potential protective agent against the harmful effects of oxidative stress.

This analysis revealed statistical data on Cytochrome c (CytC) gene expression, which plays a critical role in the intrinsic apoptosis pathway by triggering the activation of caspases that lead to programmed cell death. There was no statistically significant difference in CytC expression between the control and H_2_O_2_-treated groups (*p* = 0.1616). However, a modest increase in CytC expression was observed in the H_2_O_2_ group, which may be attributed to elevated H_2_O_2_ levels within the mitochondria due to oxidative stress. This suggests that while the oxidative stress was not severe enough to trigger significant CytC release, it may have impacted mitochondrial function. The comparison between the control and 250 µM CA-treated groups showed no statistically significant difference (*p* = 0.9431). The difference between the control and 500 µM CA-treated groups was also insignificant (*p* = 0.9953). These findings indicate that both concentrations of CA resulted in CytC expression levels like the control group. The difference in CytC expression between H_2_O_2_-treated and 250 µM CA-treated groups was not statistically significant (*p* = 0.3340). There was no statistically significant difference in expression between the H_2_O_2_-treated and 500 µM CA-treated groups (*p* = 0.1173). However, the fact that the 500 µM CA group showed CytC expression levels like the control group suggests that CA at this higher concentration may help stabilise apoptotic responses and potentially exert a protective effect on cells. The comparison between 250 µM and 500 µM CA-treated groups showed no statistically significant difference in CytC expression (*p* = 0.8598), indicating that increasing the CA dose does not result in significant changes in CytC expression. These findings suggest that while H_2_O_2_-induced oxidative stress might lead to a slight increase in CytC levels, possibly due to elevated mitochondrial H_2_O_2_, this increase does not reach levels that would trigger apoptosis. Moreover, the similarity in CytC expression between the CA-treated groups and the control group suggests that CA may play a role in maintaining cellular homeostasis and offering potential protection against the harmful effects of oxidative stress. These protective effects of CA might be mediated through mechanisms other than the intrinsic apoptotic pathway involving CytC.

**Figure 4:**
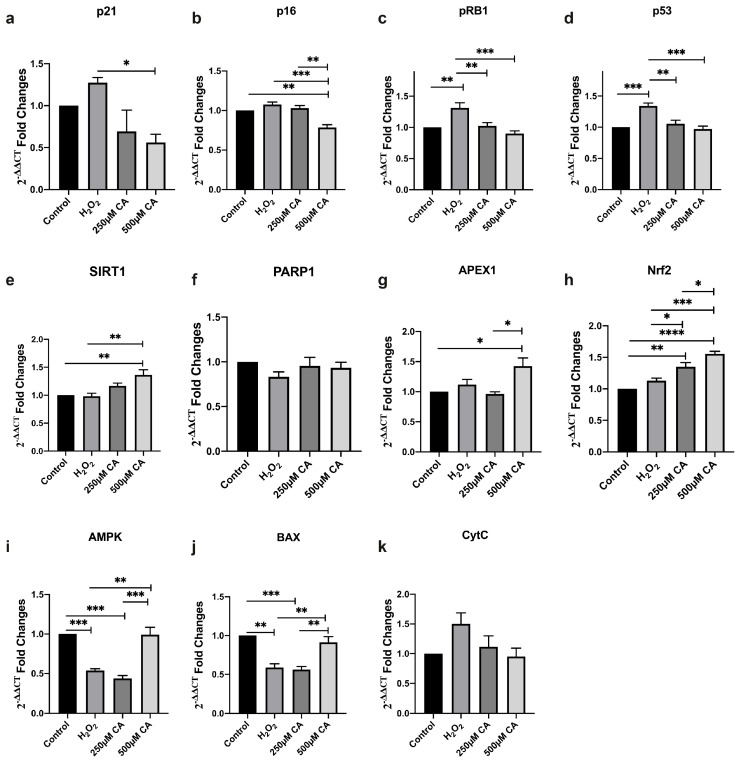
Gene expression analysis was quantified by RT-qPCR in BJ fibroblast cells. RT-qPCR analysis of the target genes normalised to the β-actin reference gene. All target genes’ overall expression levels are compared with the reference gene. Statistical analysis was conducted using one-way ANOVA, followed by Tukey’s test to determine significant differences (* *p* < 0.05, ** *p* < 0.01, *** *p* < 0.001, **** *p* < 0.0001). This figure illustrates the fold change in gene expression for p21 (**a**), p16 (**b**), pRB1 (**c**), p53 (**d**), SIRT1 (**e**), PARP1 (**f**), APEX1 (**g**), Nrf2 (**h**), AMPK (**i**), BAX (**j**), and CytC (**k**).

Fold change in gene expression analysis showed changes in SIPS and CA-treated groups compared to control. However, these changes were relatively modest, for most genes show fold changes between 1- and 1.5-fold. In SIPS models, the induced stress is controlled to prevent cytotoxicity while promoting senescence. This controlled nature usually leads to mild rather than severe changes in gene expression. Therefore, fold changes in gene expression may be lower than in a more acute stress or damage model. This finding suggests that, although CA treatment affects gene expression associated with DNA damage repair and oxidative stress response, these changes remain modest. These results are consistent with the proposed SIPS assessment (Figure 4).

#### 3.5.2. Protein Analysis Results

**Figure 5:**
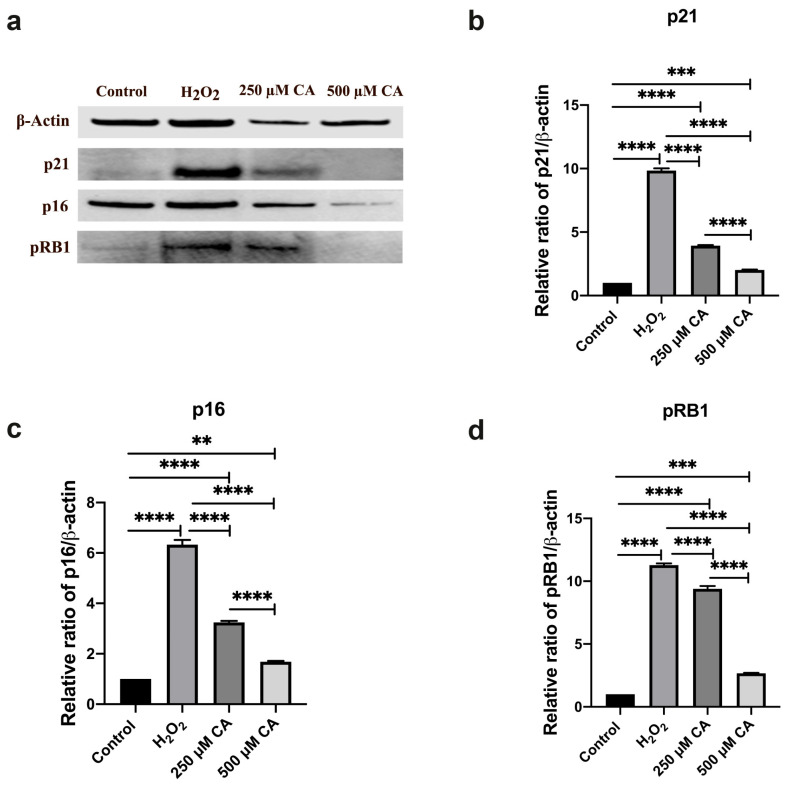
(**a**) Representative western blot images display the protein levels across four distinct groups: untreated control, cells treated with H_2_O_2_, and cells treated with 250 µM and 500 µM of CA. (**b**) Densitometric analysis of p21 protein levels. (**c**) Densitometric analysis of p16 protein levels. (**d**) Densitometric analysis of pRB1 protein levels. Protein levels were normalised to β-actin. Data are means ± SEM from independent experiments. Values are analysed using GraphPad Prism software through the one-way ANOVA and Tukey’s Post hoc assay. Statistical significance is indicated as ** *p* < 0.01, *** *p* < 0.001, and **** *p* < 0.0001; differences are relative to the control group. This figure highlights the effects of H_2_O_2_ and CA treatments on protein expression.

Since small mRNA expression changes can significantly impact protein expression, three senescence marker genes (p21, p16 and pRB1) with significant expression values at the mRNA expression level in comparison to the control group were also evaluated at the protein level. To ensure accurate quantification, β-actin was used as the housekeeping protein in our western blot analyses. The ImageJ software was used to analyse Western blot images. Western blotting revealed elevated SIPS-associated p21, p16 and pRB1 protein levels compared to the control group. This increase was visually supported by the markedly intensified bands in the groups exposed to oxidative stress (Figure 5a). Furthermore, the densitometric bar graphs in Figure 5b–d show quantitative changes in protein expression over time; distinct peak heights correspond to stress conditions.

The effect of chelidonic acid treatment on p16 protein expression was also evaluated. The results showed a statistically significant increase in p16 protein levels in the H_2_O_2_-treated group than the control group (*p* < 0.0001). This finding indicated that oxidative stress induced p16 protein expression. A statistically significant difference was also observed between the control group and both the 250 µM CA (*p* < 0.0001) and 500 µM CA (*p* = 0.005)-treated groups, suggesting that the CA can modulate p16 protein levels in the absence of oxidative stress.

Moreover, the differences between the H_2_O_2_ group and the 250 µM CA (*p* < 0.0001) and 500 µM CA (*p* < 0.0001) groups were statistically significant, indicating that CA can effectively reduce the stress-induced upregulation of p16. The fact that the 500 µM CA group exhibited significantly lower p16 protein levels than the 250 µM CA group (*p* < 0.0001) emphasises the dose-dependent effect of CA in downregulating p16 expression. These findings suggested that oxidative stress increases p16 protein levels. Chelidonic acid exerts a protective effect by downregulating p16 protein levels, potentially mitigating the senescence markers. The double modulation of p16 at the gene and protein levels highlights CA’s potential as a therapeutic agent in managing cellular senescence and related pathologies, mainly through influence on the p16 pathway.

Similarly, the effect of chelidonic acid treatment on p21 protein expression was evaluated. The difference between the control and H_2_O_2_ groups was statistically significant (*p* < 0.0001), indicating that oxidative stress upregulates p21 at the protein level, although no significant change at the mRNA level. This finding suggests a post-transcriptional regulation mechanism by which p21 protein stability or translation increases under oxidative stress conditions, leading to accumulation. A statistically significant difference was also found between the control group and 250 μM CA (*p* < 0.0001) and 500 μM CA (*p* = 0.0002). In addition, the differences between H_2_O_2_ and 250 μM CA (*p* < 0.0001) and 500 μM CA groups (*p* < 0.0001) were statistically significant, indicating that CA can effectively counteract the oxidative stress-induced increase in p21 protein. The difference between 250 µM CA and 500 µM CA groups was statistically significant (*p* < 0.0001). The fact that the 500 µM CA group showed lower p21 protein levels than the 250 µM CA group (*p* < 0.0001) emphasises that CA has a dose-dependent effect in reducing p21 protein expression, which agrees with the observed gene expression tendencies. It significantly upregulates p21 at the protein level, possibly through post-transcriptional mechanisms. These findings suggest that oxidative stress does not significantly alter p21 gene expression. Chelidonic acid exerted a protective effect by downregulating p21 protein levels, especially at high concentrations, which may alleviate the onset of premature senescence. This double modulation of p21 at both gene and protein levels highlights its potential as a therapeutic agent in managing cellular senescence and related pathologies, mainly through modulation of the p53-p21 pathway.

Lastly, the effect of chelidonic acid (CA) treatments on pRB1 protein expression was evaluated. The Tukey test revealed a statistically significant increase in pRB1 protein levels in the H_2_O_2_-treated group than the control group (*p* < 0.0001), confirming that oxidative stress upregulated pRB1 protein. There were statistically significant differences in pRB1 protein levels between the control group and both the 250 µM CA-treated group (*p* < 0.0001) and the 500 µM CA-treated group (*p* = 0.0001), suggesting that the CA treatment impacted pRB1 protein levels. CA treatments significantly reduced the oxidative stress-induced increase in pRB1 protein levels. The 250 µM CA-treated group (*p* < 0.0001) and the 500 µM CA-treated group (*p* < 0.0001) showed significantly lower pRB1 protein levels than the H_2_O_2_-treated group, indicating that the CA can mitigate the stress-induced upregulation of pRB1. Furthermore, the difference in pRB1 protein levels between the 250 µM and 500 µM CA-treated groups was statistically significant (*p* < 0.0001), emphasising a dose-dependent effect of CA in modulating pRB1 protein expression. These findings suggest that chelidonic acid effectively downregulates pRB1 protein expression in response to oxidative stress, particularly at higher concentrations. The CA may help prevent stress-induced permanent cell cycle arrest, highlighting its potential therapeutic role in managing conditions, including oxidative stress and senescence.

## 4. Discussion

This study is the first to explore the effects and mechanisms of action CA on H_2_O_2_-induced premature senescence in BJ fibroblast cells. CA treatment was found to effectively reduce cellular senescence in a dose-dependent manner. The underlying mechanisms are linked to the regulation of critical senescence-related pathways, including p53, pRB1, and SIRT1. Our findings highlight the potential of CA to function as a senomorphic and senolytic agent.

Chelidonic acid, a secondary plant metabolite, exhibits important pharmacological properties that mitigate or reverse cellular senescence. CA’s anti-inflammatory properties can slow the senescence process by reducing cellular stress and improving the cellular environment [76]. CA has been shown to reduce the levels of pro-inflammatory cytokines, such as IL-6 and TNF-α, contributing to the senescence process and cellular senescence [76]. Additionally, studies have demonstrated that CA mitigates oxidative stress damage by reducing the expression of cyclooxygenase-2 (COX-2) [45], and CA increases the hypoxia-inducible factor-1a (HIF-1a) [77]. This finding indicates that CA is an important compound that helps combat cellular senescence. Consistent with the findings of previous studies, our data suggest that CA acts on the underlying mechanisms that trigger senescence and may be helpful in therapies to prolong cellular health and combat age-related diseases.

Premature senescence is a cellular mechanism that requires careful calibration of the experimental conditions. This avoids excessive damage that might trigger necrosis or apoptosis and ensures that the observed effects are specific to the senescence pathway. Fine-tuning oxidative stress to the minimum adequate level can illuminate the threshold at which oxidative damage pushes cells towards senescence, thereby elucidating the delicate balance between stress and survival in cellular senescence [46]. Based on the literature data, early-stage cells were used in all experiments to prevent the appearance of replicative senescence. The key characteristics of both replicative and stress-induced senescence are known to be similar [78]. A growth curve was constructed for the BJ fibroblast cell line to investigate the growth parameters and determine the optimal conditions for inducing premature senescence through growth rate optimisation. This curve facilitated the acquisition of critical metrics such as population doubling time, lag time, and saturation density; these data are instrumental in understanding and triggering senescence (Figure 1a). 

Beta-galactosidase staining, which emerged as a marker for SCs following the work of Hayflick and Moorhead, plays a crucial role as an indicator of replicative senescence [79,80]. Traditionally, in vitro fibroblast cultures undergo a phase of rapid growth (IIa) before a slowdown (IIb), which eventually leads to phase III, where cells enter a senescent state [81]. Although the use of beta-galactosidase activity at pH 6 as a senescence biomarker has been debated because of its upregulation under various stress conditions, its strong correlation with cellular growth rate has been confirmed in multiple studies [82]. The H_2_O_2_-induced premature senescence experimental model in BJ fibroblast cells required selecting a time point that minimised replicative senescence. The data (Figure 1b) demonstrated an apparent time-dependent increase in SA-β-Gal activity, a widely accepted biomarker of cellular senescence [83]. The progression from day 3 to day 9 indicated that BJ fibroblast cells underwent intrinsic senescence without external stressors (Figure 1b,e). Oxidative stress was applied early in the growth curve, particularly before the transition from phase IIa to IIb.

It was crucial to determine the sublethal dose range of H_2_O_2_ for subsequent studies. Cell viability was assessed using the MTT assay [84]. The viability of BJ fibroblast cells decreased in a dose-dependent manner in response to H_2_O_2_ (Figure 1d). Previous research by Zhu et al. demonstrated that sublethal concentrations of H_2_O_2_ (<300 µM) can induce growth arrest and promote cellular senescence without adversely affecting the survival of human diploid fibroblasts [85]. Our results showed no significant 50–600 µM H_2_O_2_-treatment effect on cell viability. Next, the effect of CA on BJ fibroblast cell proliferation and viability was evaluated using the MTT reduction assay [47]. Our results revealed that the effect of CA on cell viability depended on its concentration and exposure time (Figure 1e). Consistent with previous studies, it was noted that concentrations up to 1000 μM were not cytotoxic. Our findings suggest that CA has the potential to modulate cell growth without toxicity in clinical and research settings.

One of the main aims of this study was to create a model for SIPS. The oxy-inflamm hypothesis suggests that oxidative stress, which increases with age, significantly contributes to the onset of senescence [86]. H_2_O_2_, widely recognised for its role in inducing oxidative stress in various cell types [87], was used at a sublethal dose for the minimum time required for SIPS.

As reported by Kumar et al. treatment with sublethal concentrations of H_2_O_2_ induces cellular senescence (Figure 2a and Figure 3a) [88]. This finding is evidenced by typical senescence-associated features, such as increased cell size and the development of SASP, including increased SA-β-gal expression. The ability of CA treatment on BJ fibroblast cells after SIPS was highlighted. The capacity of CA to decrease the number of SA-β-Gal-positive cells highlights its ability to regulate the stress response and contribute to the repair of cells after oxidative damage (Figure 2a and Figure 3a). A clinical study reported that a combination of dasatinib and quarcetin reduced the number of SA-β-gal activity-positive cells [89]. The senomorphic agent metformin treatment has been reported to reduce SA-β-gal activity in cells [90,91]. In addition, the research findings are consistent with previous studies that found bioactive plant compounds such as caffeic acid, quercetin, and resveratrol ameliorated oxidative stress-induced senescence and promoted cell proliferation in various cell types [28,92,93].

The results prompted us to investigate whether exposure to H_2_O_2_ impacted the structure of the cell nucleus. Consistent with our findings, Gerasymchuk et al. noted that young fibroblasts typically display an elongated, spindle-like morphology and adhere firmly to culture plates. On the other hand, senescent fibroblasts, or those treated with H_2_O_2_, tend to be larger, irregularly shaped, and less frequent [46]. Fibroblasts were stained with DAPI, a fluorescent dye with an affinity for DNA sequences high in adenine and thymine [94]. In cells that had only been exposed to H_2_O_2_, nuclear DAPI staining showed more nuclear atypia, which includes chromatin condensation and changes in shape, like nuclear enlargement or irregularities. These changes indicate cellular stress or senescence [25,95]. Interestingly, CA treatment significantly reduced these nuclear alterations in cells. This finding suggests that CA might play a role in safeguarding nuclear integrity and stability in the face of oxidative stress (Figure 2b and Figure 3b). Furthermore, acridine orange staining (AO) was used to assess the lysosomal activity of the cells [96]. This activity can be heightened during senescence as the cell attempts to recycle damaged proteins and organelles. Both the number and fluorescence intensity of AO-stained cells were lower in the CA-treated groups than in the H_2_O_2_ group, suggesting an effect of CA on lysosomal activity and DNA damage (Figure 2c and Figure 3c). Consistent with our results, other researchers have shown that cellular lysosomal content increases during senescence [97]. In vitro senescence has been linked to increased levels of lysosomal enzymes [98]. Kurz et al. reported that SCs are large and filled with numerous lysosomes [99].

Alkaline comet assay was used to evaluate DNA damage in all groups (Figure 2d). The control, SIPS group, and CA treatment groups showed differences in the size of the nuclear cells (Figure 2d and Figure 3d). Control group cells exhibited uniformly small nuclei with a regular, rounded morphology. On the other hand, the SIPS group exhibited a mixture of both small and large nuclei and variations in nuclear shape. An uneven fluorescence distribution distinguished the senescent cultures. CA-treated groups exhibited nuclear morphology closer to the control group, and the effect was dose-dependent. Our findings are consistent with the studies in the literature [100].

Oxidative stress is known to have many detrimental effects on critical cellular components and can lead to conditions such as lipid peroxidation. While investigating CA’s ability to attenuate and reverse such oxidative stress-induced cellular senescence, it was observed that it enhances cellular antioxidant defence systems in a dose-dependent manner. This finding was evidenced by a decrease in cellular H_2_O_2_ levels (Figure 3e) and MDA levels (Figure 3f), an increase in total SOD (T-SOD) activity (Figure 3g), and maintenance of GSH homeostasis (Figure 3h). The role of oxidative stress in cellular senescence and its link with lipid peroxidation have been reported by Morliere et al. [101]. Panieri et al. reported that mitochondrially derived H_2_O_2_ induced G1 phase cell cycle arrest, while low exogenous H_2_O_2_ caused G2/M phase accumulation. These findings suggest that mitochondrial mechanisms and intracellular H_2_O_2_ levels play essential roles in cell cycle regulation and DNA damage response [102]. In an in vivo study, chelidonic acid was found to have similar effects to our study on MDA levels, SOD and GSH activities [77]. Zhang et al. reported that SCs levels were significantly increased in the kidneys of young (6-month-old) Sod1-/- mice compared to young wild-type (WT) mice [103]. GSH balance, a vital antioxidant, is also critical in maintaining cellular redox homeostasis. The senescence process is typically associated with increased oxidative stress and GSH turnover. This cell response attempts to stabilise the oxidative status, which can lead to exhaustion or decreased efficiency in restoring balance over time [104]. CA’s protective effects against oxidative stress and its role in maintaining antioxidant defence suggest that it may be an essential player in anti-senescence therapy. The results indicate that CA may be an effective compound for combating age-related functional decline.

The findings were supported by using molecular docking predictions to investigate the interaction between the compound and the target protein. Molecular docking showed a strong binding between the core compound and important targets and their anti-senescence potential. It also strengthened the relationship between CA and targets. Senescence-related molecular docking studies like our research are available in the literature [105].

RT-qPCR was employed to assess gene expression, using β-actin as a reference due to its stability across conditions [106]. The results indicate that CA modulates key senescence-related pathways, suggesting its potential in senescence therapy. Many studies have documented the phenotypical and transcriptional heterogeneity of SCs using both in vitro and in vivo models [107]. The diversity of cell types undergoing senescence significantly influences this heterogeneity. Furthermore, transcriptomic profiling reveals the expression of different genes at various stages of the senescence process. These findings underscore the complexity and variability of cellular senescence based on cellular context and gene expression timing [108]. The target genes analysed are intrinsic to various pathways involved in cellular senescence: pRB1, p53, p16, and p21 regulate cell cycle arrest, the hallmark of senescence, through the RB and p53 signalling pathways. In addition, pRB1, p16 and p21 genes associated with these two pathways were evaluated at protein level. The data suggests that p21 initiates senescence while p16 maintains it. Likely, these cyclin-dependent kinase inhibitors are sequentially involved in cellular senescence [109,110]. As reported in other studies, both major senescence pathways have been detected to be activated: the p53-p21 pathway (Figure 4a,d) and the p16-pRB1 pathway (Figure 4b,c) [5,111]. Kamal et al. reported that treatment with H_2_O_2_ induced a delayed increase in the p53 protein level. During senescence, p53 activates the transcription of the cyclin-dependent kinase inhibitor (CDKi) p21 [112]. This process inhibits CDK2 activity, resulting in the hypophosphorylation of Rb and leading to cell cycle arrest [113,114]. The gene expression and protein levels of p53 and p21 showed significant differences between the treatment groups. At the same time, the H_2_O_2_-treated group exhibited an increase in p21 gene expression than the control group. It exhibited a statistically significant increase in protein level. This finding indicates an activation of p21 in response to oxidative stress and its stable expression at the protein level. On the other hand, the 250 µM and 500 µM CA-treated groups exhibited lower expression than the H_2_O_2_-treated group. That finding suggests a regulatory or protective effect of CA against SIPS. p53 gene expression was also significantly elevated in the H_2_O_2_-treated group compared to the control group, reflecting a stress response. In the 250 µM and 500 µM CA groups, there was a significant decrease in p53 gene expression than the H_2_O_2_-treated group. This result indicates a response similar to that of the control group. Treatment with CA may modulate cell cycle arrest and oxidative stress response of the cell. Significant differences were detected between treatment groups when gene expression and protein levels of p16 and pRB1 were evaluated. Specifically, the H_2_O_2_ group exhibited a statistically significant increase in pRB1 gene expression and protein levels than the control group. While the p16 gene expression level in the H_2_O_2_ group did not show a significant increase in the control group, there was a significant difference in p16 levels between the H_2_O_2_-treated group and the control group at the protein level. These observations highlight that although p16 gene expression levels may be similar between groups, various factors can contribute to significant differences in protein levels. Such factors include post-transcriptional regulation, protein stability, translational efficiency, post-translational modification [115], and cellular context [116]. In the 250 µM and 500 µM CA groups, there was a significant decrease in pRB1 and p16 gene expressions than the H_2_O_2_-treated group. These observations highlight that pRB1 and p16 gene expression and protein levels showed significant differences between the groups and that CA may contribute significantly to reducing the expression levels of age-regulatory pRB1 and p16 proteins dose-dependently. These findings indicate that our experiment’s specific conditions concurrently activated multiple mechanisms contributing to cellular senescence. In addition, it highlights the complexity of cellular senescence and how environmental factors can influence its regulation. 

Another critical aspect of cellular senescence involves DNA repair mechanisms. Sirtuin 1 (SIRT1) and Poly (ADP-ribose) Polymerase-1 (PARP1) are DNA repair mechanisms that are part of the Sirtuin (SIRT) and Poly ADP-Ribose Polymerase (PARP) pathways, respectively [117]. SIRT1 activity is reduced in chronic inflammation and senescence conditions, where oxidative stress is familiar [118]. PARP1 is a versatile enzyme crucial in regulating specific DNA repair processes, ensuring the genome’s stability and promoting cell survival. Mice lacking PARP activity are fertile and born with no apparent health problems. However, it has been observed that radiation exposure reduces the proliferation rate of primary embryonic fibroblasts in vitro and delays the proliferation of thymocytes in vivo [119]. It has been conclusively shown that CA affects key molecular pathways associated with senescence. Specifically, 500 µM CA concentration significantly increased the expression of the SIRT1 gene than both control and H_2_O_2_-treated groups. This increase in SIRT1 expression promotes a cellular environment that is more resilient to senescence and associated cellular damage (Figure 4e). In addition, PARP1 gene expression levels remained consistent in all groups. This finding indicates that it may not have caused DNA damage at a level requiring PARP1 activation. It also suggests that the effects of CA may be selectively focused on specific pathways associated with senescence (Figure 4f).

In addition, APEX1 is part of the Base Excision Repair (BER) pathway and mainly helps to fix DNA and control redox factors that control transcription [120]. According to the gene expression results, especially 500 µM CA treatment significantly increased APEX1 gene expression compared to the control and 250 µM CA-treated groups. At the same time, There was no statistically significant increase in the H_2_O_2_ group. This finding suggests that CA can potentially enhance DNA repair mechanisms (Figure 4g). In line with these findings, the researchers also developed a hypomorphic mouse model with lower levels of APEX1 using a neomycin resistance cassette. The offspring were smaller and exhibited lower APEX1 levels, malformed tails, and increased oxidative DNA damage. These findings highlight the critical role of APE1 in development and DNA protection [121].

The Nrf2 Antioxidant Response Pathway regulates the cellular reactions to oxidative stress [122]. Because it can enhance the production of proteins that scavenge ROS and antioxidant enzymes, it is considered a crucial element in lifespan signalling pathways [123]. Suppression of Nrf2 significantly accelerates cellular senescence, highlighting the importance of Nrf2 in safeguarding against senescence [124]. Our study showed that Nrf2 gene expression levels did not significantly change in cells undergoing H_2_O_2_-induced senescence than the control group. Under these senescence conditions, treatment with CA dose-dependently increased the Nrf2 gene expression level than the control and H_2_O_2_ groups. This finding suggested that CA ameliorated Nrf2 expression (Figure 4h). Activation of the Nrf2-dependent antioxidant defence system effectively prevents senescence [125].

The AMPK pathway regulates cellular energy homeostasis [126]. When AMPK gene expression levels were evaluated, a statistically significant decrease was observed in the H_2_O_2_ group than the control group. The 500 µM CA group showed a statistically significant increase in AMPK expression levels than the H_2_O_2_ group. Treatment with 500 µM CA improved the response of the metabolic system to senescent cells by activating the AMPK-SIRT1-Nrf2 axis in BJ fibroblast cells (Figure 4i. Comparable to the research findings, Khairnar et al. reported that chelidonic acid increased AMPK levels in the in vivo Nephrotoxicity study [77]. Previous research has demonstrated that metformin can mitigate oxidative stress-induced senescence by activating AMPK in human lens epithelial cells [127]. In one study, SIRT1 and AMPK were shown to control each other mutually [128].

Cytochrome c (CytC) is a widely recognised protein that is located on the outer surface of the inner mitochondrial membrane. CytC is considered an essential regulator of oxidative stress, and it exhibits peroxidase-like activity [129]. CytC and Bax are critical components of the apoptotic machinery within the Intrinsic Apoptotic Pathway [130]. In this study, the H_2_O_2_-treated group showed significantly lower Bax levels than the control group, resulting in cell cycle arrest (Figure 4j). Furthermore, increased intracellular levels of H_2_O_2_ contributed to the upregulation of CytC expression, probably due to increased peroxidase activity. In contrast, cells treated with 500 µM CA after H_2_O_2_ exposure exhibited significantly increased Bax levels and no statistically significant difference in CytC levels than the H_2_O_2_ group, indicating selective apoptosis (Figure 4k). This response implies that CA has potential senolytic properties at this concentration. Similarly, the first senolytics blocked the ability of SCs to evade programmed cell death, mainly by inhibiting anti-apoptotic Bcl-2 family proteins [131,132]. The ability of CA to upregulate Bax and potentially promote apoptosis in damaged SCs while simultaneously reducing CytC to stabilise mitochondria and minimise unnecessary cell death highlights its therapeutic potential in senescence research. This dual mechanism highlights the complex balance between cellular survival and apoptosis pathways and positions CA as a promising therapeutic agent for managing and potentially reversing cellular senescence (Figure 4j,k).

## 5. Conclusions

Short-term H_2_O_2_ treatment is an established and widely used experimental model for inducing oxidative stress and accelerating cellular senescence. However, it is recognised that this model does not exactly replicate the natural, gradual senescence process that typically occurs over an extended time. Spontaneous senescence is a cellular stress response to cumulative damage, telomere shortening and chronic activation of DNA damage response pathways caused by various stressors. These stressors and their resulting phenotypes may not be fully reflected by H_2_O_2_-induced acute oxidative stress. This limitation may affect the applicability of the findings to more complex biological systems in which senescence develops more slowly. Nevertheless, this model allows a controlled and reproducible induction of senescence, facilitating the study of specific molecular and cellular mechanisms in response to oxidative stress. The information obtained from this model is valuable for understanding the early stage of senescence onset and the potential therapeutic effects of chelidonic acid in alleviating and reversing stress-induced cellular senescence. 

Our study revealed that chelidonic acid regulates several critical pathways associated with cellular senescence, particularly by attenuating oxidative stress markers and inhibiting and reversing the progression of senescence-related phenotypes in BJ fibroblast cells. This compound not only reduced SA-βgal activity but also effectively reduced oxidative stress markers, such as MDA and improved antioxidant defences by increasing SOD and GSH activities. Our molecular docking studies identified strong interactions between CA and proteins that play essential roles in senescence signalling pathways, suggesting that CA may interfere with cellular mechanisms that facilitate senescence. Furthermore, the consistency between our gene expression and protein analysis data supports the idea that CA may function as a senomorphic agent that reduces the effects of oxidative stress. This finding is evidenced by reductions in SA-β-Gal positive cells and changes in gene expression and protein levels associated with senescence. In addition, CA shows potential senolytic activity in vitro, suggesting that it may affect cellular senescence and oxidative stress pathways. 

Given the effect of the compound on cellular markers and the cellular microenvironment, it has significant potential in medical and cosmetic applications to manage conditions associated with senescence and oxidative stress. In medical applications, CA shows potential in the treatment of age-related diseases and conditions exacerbated by oxidative stress, such as cardiovascular diseases and neurodegenerative disorders. In comparison with senolytic agents such as Dasatinib, Quercetin and Navitoclax and senomorphic agents such as Metformin, Rapamycin and Resveratrol, the dual senomorphic and senolytic potential of CA with similar mechanisms of action suggests that it may offer complementary benefits. In cosmetic applications, CA can be used as an active ingredient in external skin compositions for anti-senescence effects, such as skin elasticity, moisturising, skin regeneration, and skin wrinkle improvement. CA can be combined with various active compounds, such as collagen and hyaluronic acid, among others, to increase its effectiveness and expand its applications in cosmetic formulations. The properties of CA, such as its small pyron ring and low toxic effect, enable it to be absorbed transdermally in effective doses and be used effectively in cosmetic applications due to its low side effects. The promising results of this study encourage follow-up long-term in vitro studies and in vivo investigations to confirm and extend understanding of the mechanisms and efficacy of CA in a comprehensive senescence-related context. More research is needed to compare CA to novel cosmetic ingredients such as antioxidant Resveratrol, anti-inflammatory Cannabinoids, and Retinols with moisturising effects. Future research should focus on improving the bioavailability and delivery mechanisms of CA to enhance its practical application in clinical settings.

## Figures and Tables

**Table 1:** Antibodies were used in this study.

Antibodies	Source	Catalog
Anti-p16 polyclonal antibody	Proteintech	10883-1-AP
Anti-p21 polyclonal antibody	Proteintech	10355-1-AP
Anti-pRB1 monoclonal antibody	Proteintech	67521-1-16
Anti- β-Actin Rabbit mAb	ABclonal	AC026
HRP-conjugated Goat anti-Rabbit IgG (H + L)	ABclonal	AS014
HRP-conjugated Goat anti- Mouse IgG (H + L)	ABclonal	AS071

**Table 2:** Molecular Docking Parameters.

Number	Complexes	PDB Code	Docking Scores (kcal/mol)	Types of Interactions	Interacting Residues
1	P21-Chelidonic acid	5XVG [63]	−12.68	Conventional H-bonding	LEU 475. TYR 492, VAL 476, LYS 473
2	P16-Chelidonic acid	1GIH [64]	−11.99	Conventional H-bonding, π Anion, π Sigma	ARG 126, VAL 164, THR 165, THR 14, ASP 127, ARG 169, LYS 129
3	P53-Chelidonic acid	4MZI [65]	−10.13	Conventional H-bonding, π Alkyl	LYS 132, ARG 273, PRO 250, ARG 248, SER 240
4	PRB1-Chelidonic acid	1AD6 [66]	−6.81	Van der waals, Conventional and Carbon H-bonding, π Alkyl	ASN 390, SER 499, ARG 445, ARG 500,
5	NRF2-Chelidonic acid	3WN7 [67]	−7.71	Conventional and Carbon H-bonding	Val 463, ILE 416, ARG 415, VAL 465, GLY 511, GLY 509
6	NF-κB-Chelidonic acid	1SVC [68]	−7.89	Van der waals, Conventional and Carbon H-bonding, π Alkyl	ARG 57, SER 243, ASN 250, LYS 244
7	JAK1-Chelidonic acid	4L00 [69]	−11.31	Van der waals, Conventional and Carbon H-bonding, π Alkyl	ARG 577, ARG 643
8	JAK2-Chelidonic acid	4GL9 [70]	−7.17	Conventional and Carbon H-bonding, π Cation, π Sigma	LYS 1009, GLU 1006, LYS 1005, LYS 1030
9	AMPK-Chelidonic acid	7OPM [71]	−7.72	Conventional and Carbon H-bonding	GLU 186, LEU 184, LYS 203
10	STAT3-Chelidonic acid	5AX3 [72]	−6.94	Van der waals, Conventional H-bonding	LYS 221, THR 181, LYS 142
11	HSP90-Chelidonic acid	6KSQ [73]	−7.68	Van der waals, Conventional and Carbon H-bonding, π Alkyl	THR 425, SER 476, GLU 461, THR 425, ALA 428, LYS 478, ARG 464
12	FOXO4-Chelidonic acid	3L2C [74]	−7.09	Conventional H-bonding	Glu 125, ARG 429

## Data Availability

Data supporting the findings of this study are available from the corresponding author upon request.

## Supplementary Materials

The following supporting information can be downloaded at: https://www.mdpi.com/article/10.3390/life14091070/s1, Figure S1: 2D and 3D poses obtained from molecular docking interaction models of Chelidonic acid (CA) with 12 target proteins. Hydrogen bonds, hydrophobic interactions, and other bonds (van der Waals, π-sigma, π-alkyl, bumps) formed between CA and amino acid residues in the active sites of proteins are illustrated. According to the docking scores, CA successfully forms complexes with all proteins. These findings are consistent with the results of in vitro experiments. Table S1: Primer sequences used for gene expression analysis in BJ fibroblast cell line. This table lists the primer sequences designed for quantitative real-time PCR analysis of various senescence-related genes, including pRB1, p53, p16, p21, SIRT1, PARP1, AMPK, NRF2, APEX1, Bax, Cytochrome C and β-Actin (control gene). The table includes gene names, primer sequences, %GC content and melting temperatures (Tm), providing basic details for the gene expression analysis performed in this study.
