# Supplementary material for: Investigating the Effects of Chelidonic Acid on Oxidative Stress-Induced Premature Cellular Senescence in Human Skin Fibroblast Cells
Source: Life (Basel). 2024 Aug 27;14(9):1070. doi: 10.3390/life14091070 (PMC11433492; doi:10.3390/life14091070)

1.P21 (5XVG)

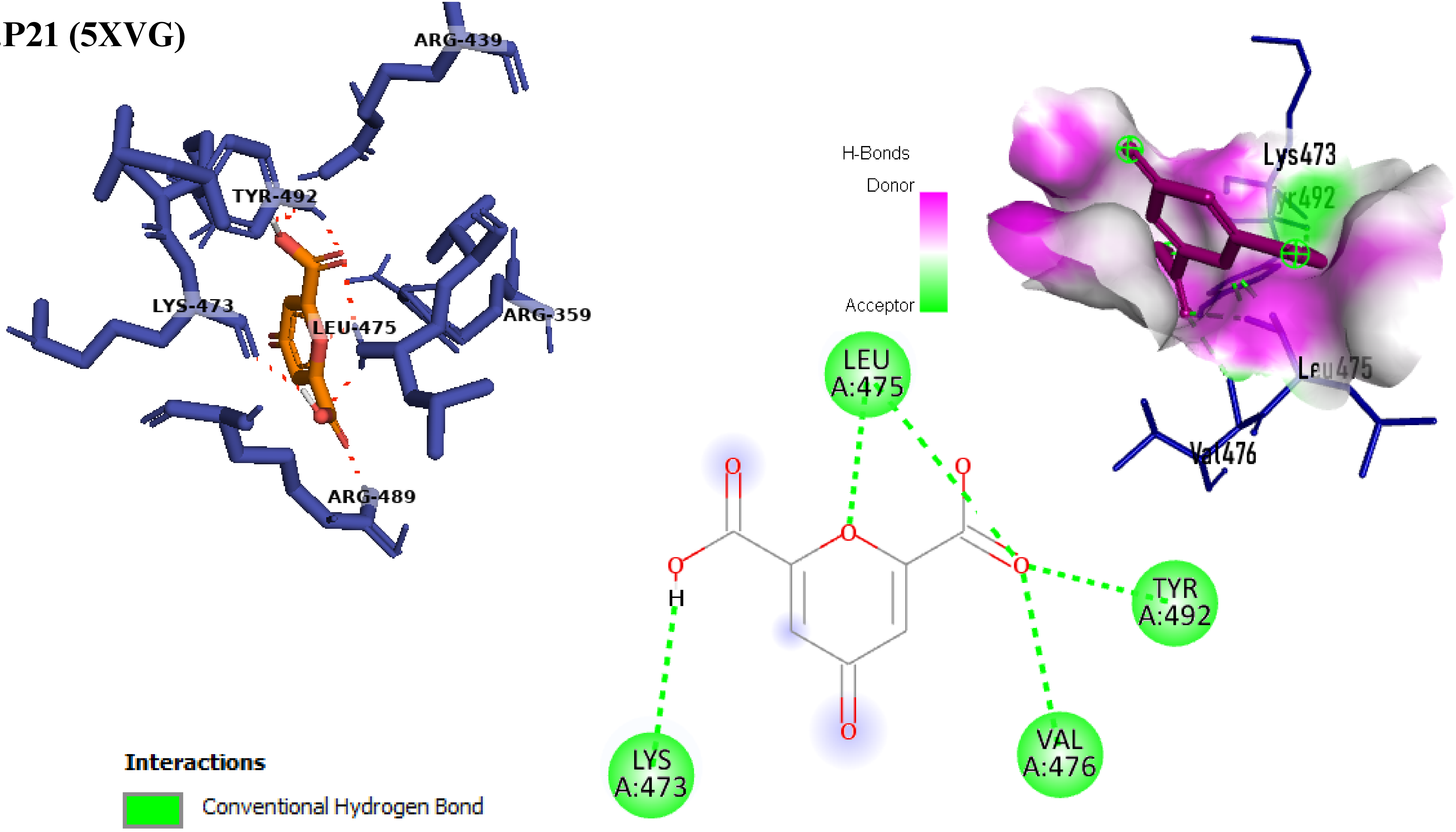
ARG-439
TYR-492
LYS-473
LEU-475
ARG-359
ARG-489
Interactions
Conventional Hydrogen Bond
H-Bonds
Donor
Acceptor
LEU A:475
TYR A:492
VAL A:476
LYS A:473
Lys473
Tyr492
Leu475
Val476

2. P16 (1GIH)

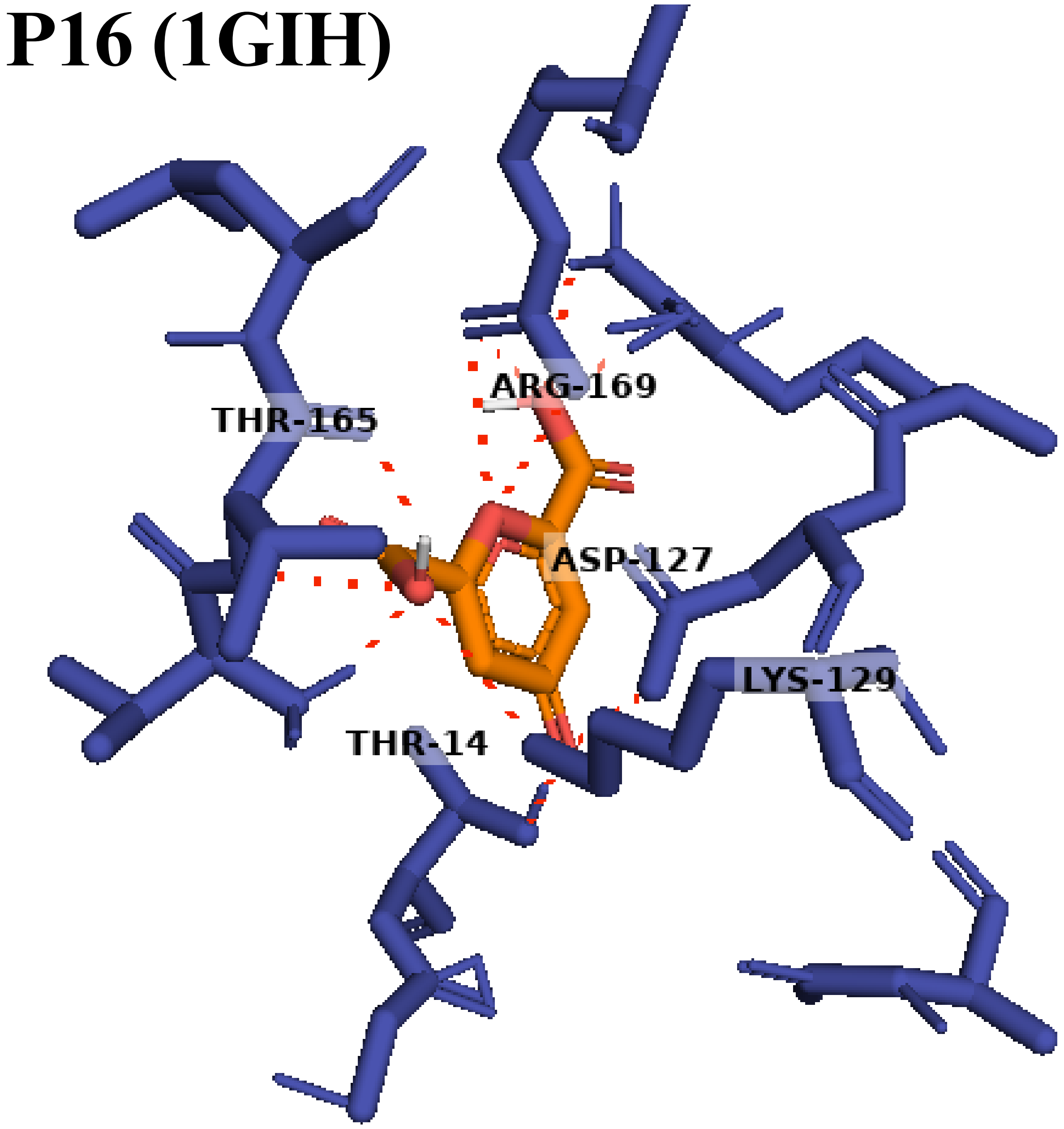

THR-165
ARG-169
ASP-127
LYS-129
THR-14

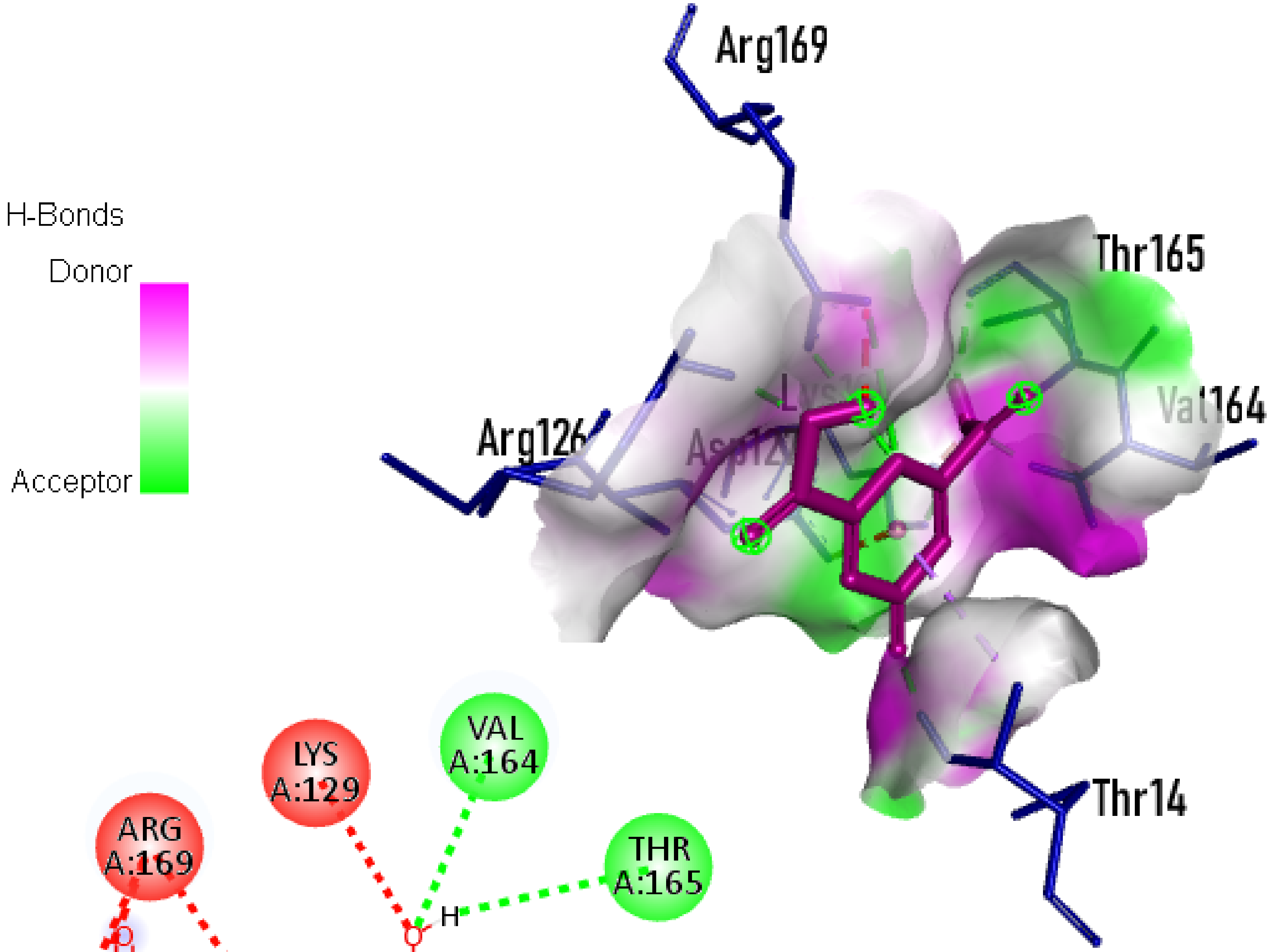

H-Bonds
Donor
Acceptor
Arg169
Thr165
Arg126
Val164
Thr14

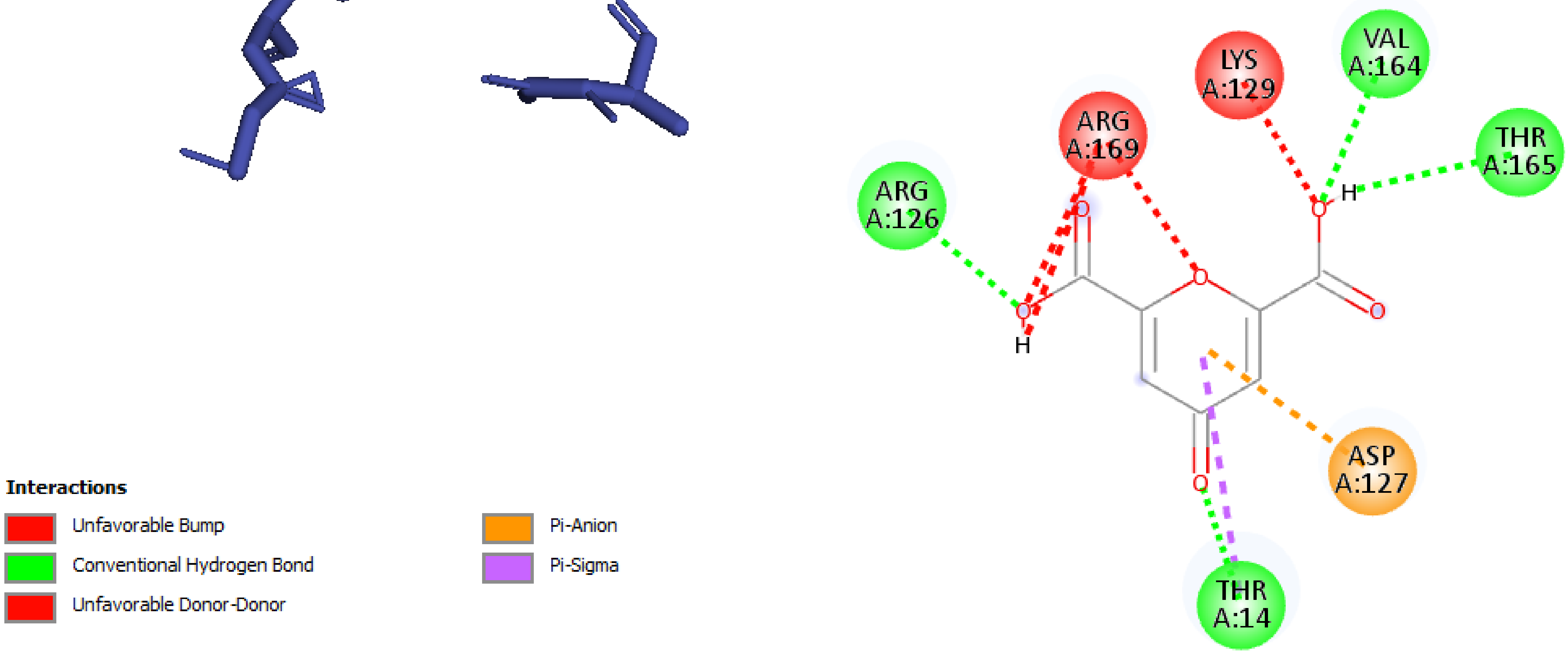

ARG A:126
ARG A:169
LYS A:129
VAL A:164
THR A:165
ASP A:127
THR A:14
Interactions
Unfavorable Bump
Conventional Hydrogen Bond
Unfavorable Donor-Donor
Pi-Anion
Pi-Sigma

3. P53 (4MZI)

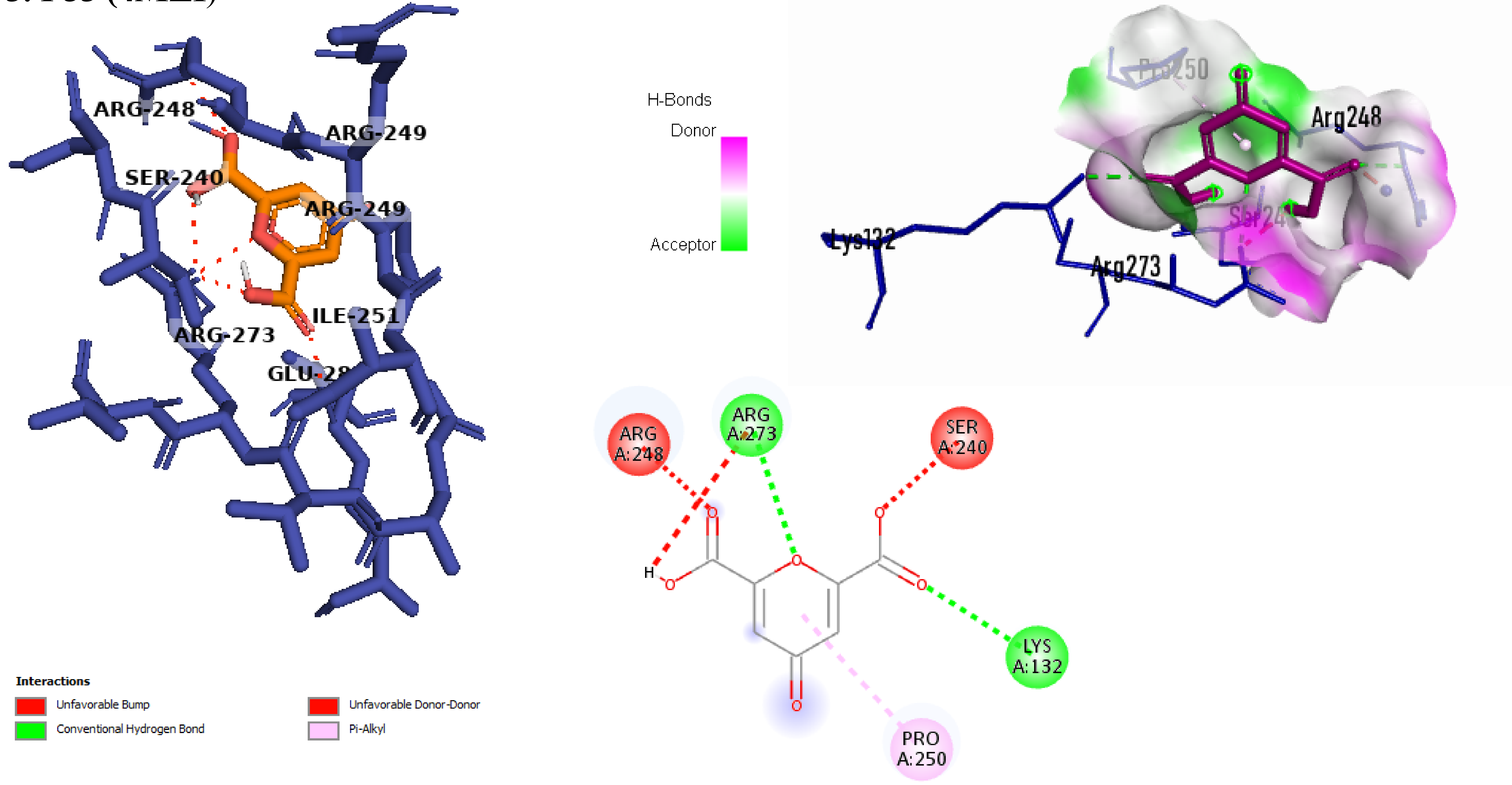

ARG-248
ARG-249
SER-240
ARG-249
ILE-251
ARG-273
Interactions
Unfavorable Bump
Conventional Hydrogen Bond
Unfavorable Donor-Donor
Pi-Alkyl
H-Bonds
Donor
Acceptor
Arg248
Lys132
Arg273
ARG A:248
ARG A:273
SER A:240
LYS A:132
PRO A:250

4. PRb (1AD6)

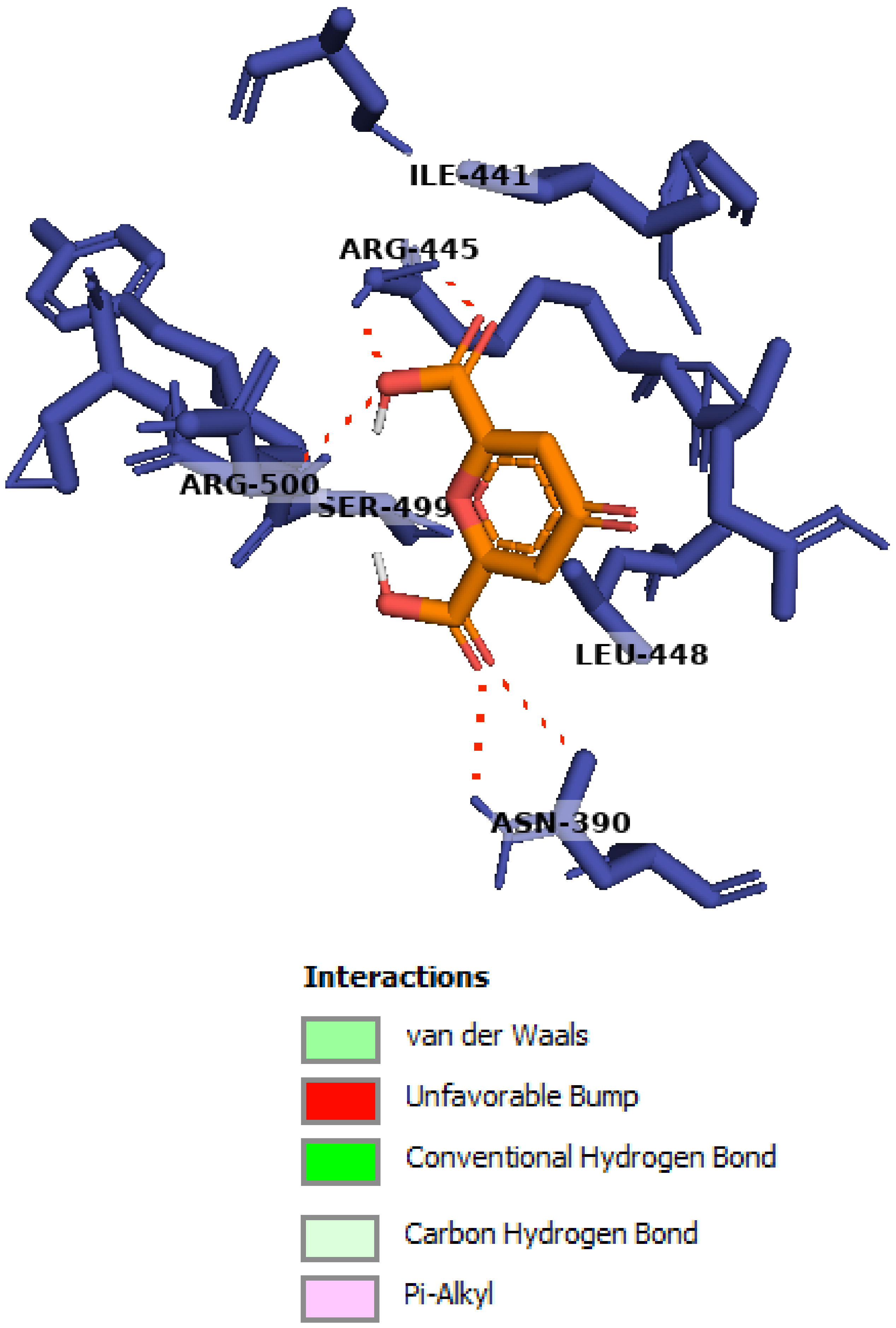
ILE-441
ARG-445
ARG-500
SER-499
LEU-448
ASN-390
Interactions
van der Waals
Unfavorable Bump
Conventional Hydrogen Bond
Carbon Hydrogen Bond
Pi-Alkyl

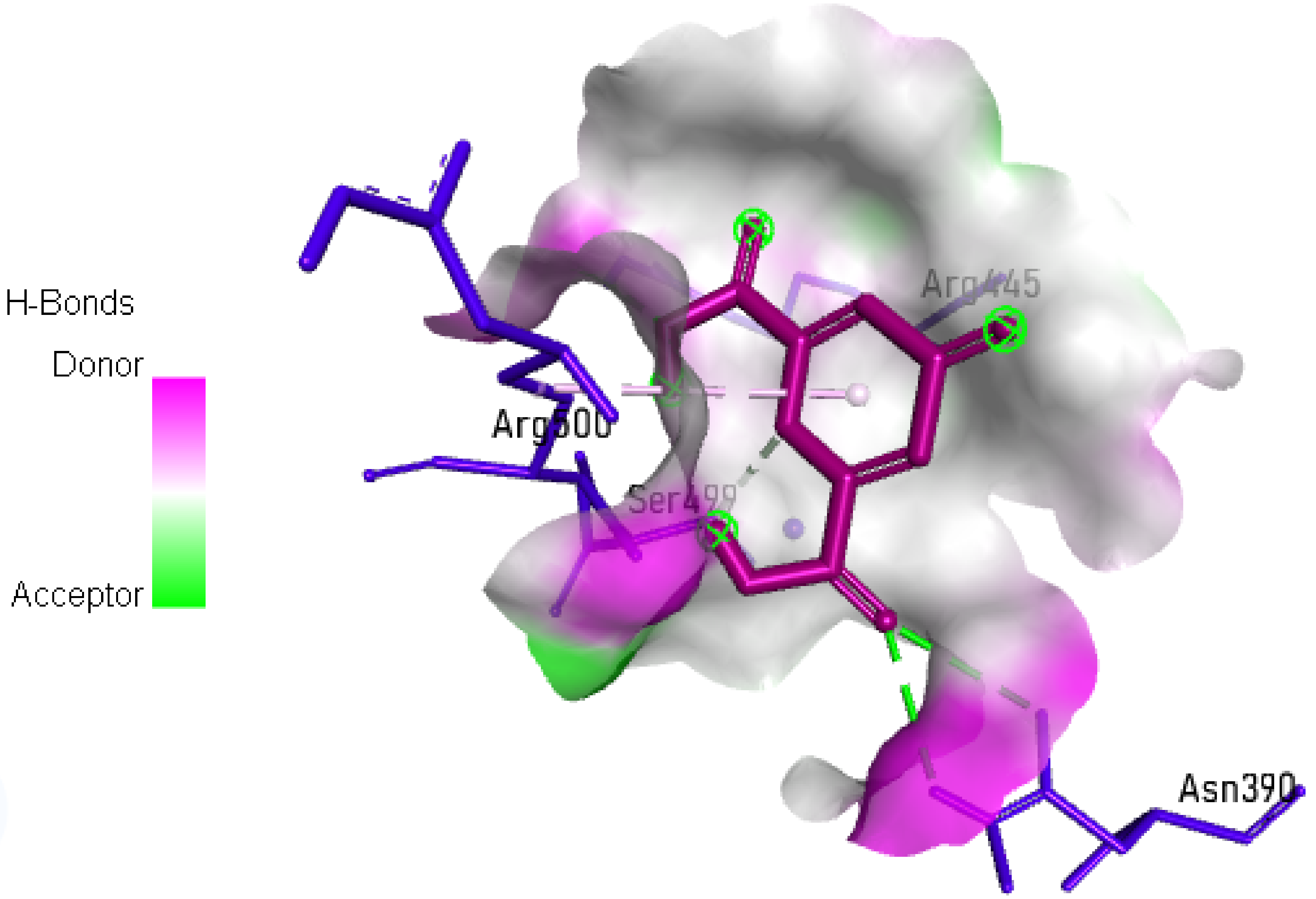
H-Bonds
Donor
Acceptor
Arg445
Arg500
Ser499
Asn390

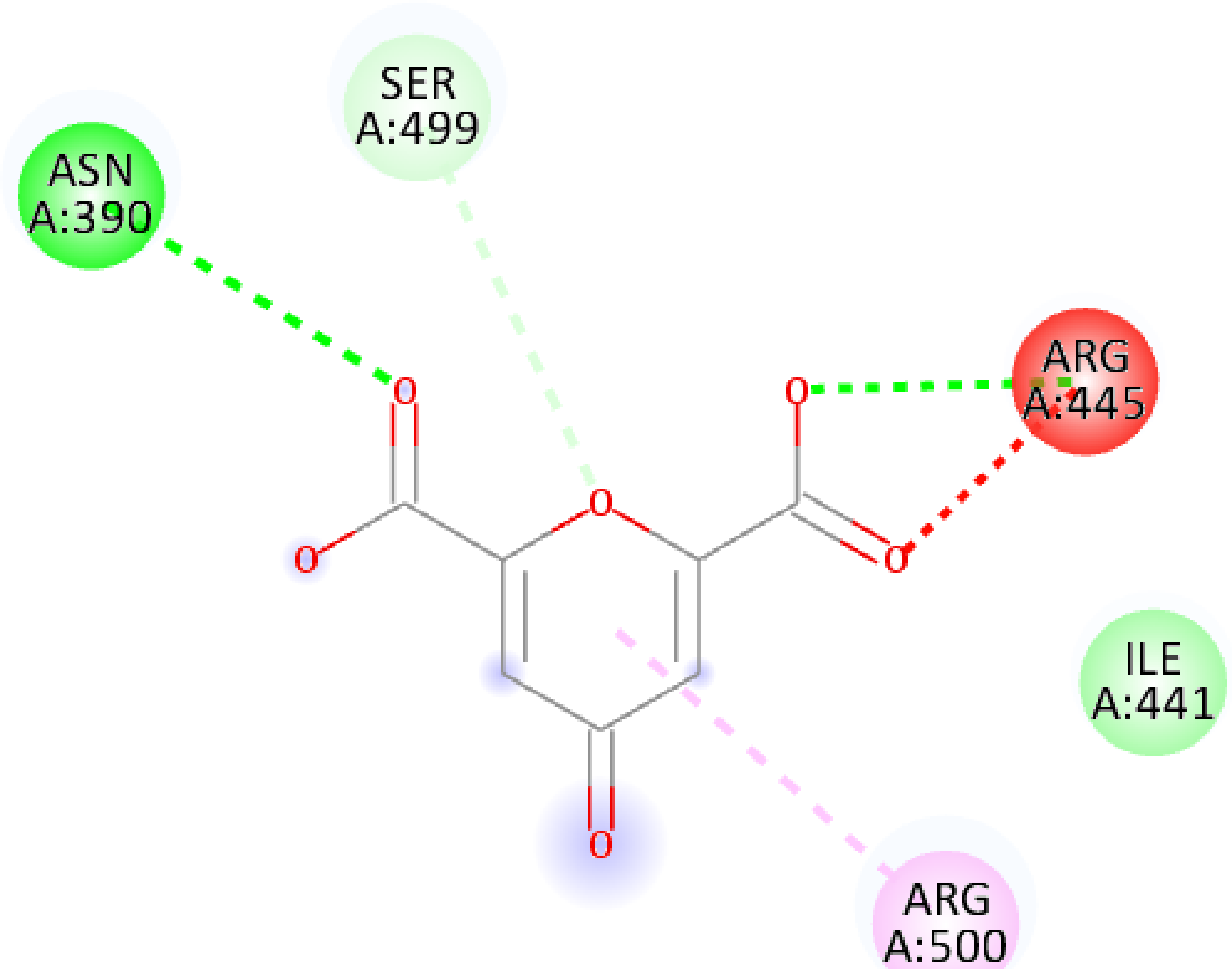
SER
A:499
ASN
A:390
ARG
A:445
ILE
A:441
ARG
A:500
O
O
O
O
O
O

5. NRF2 (3WN7)

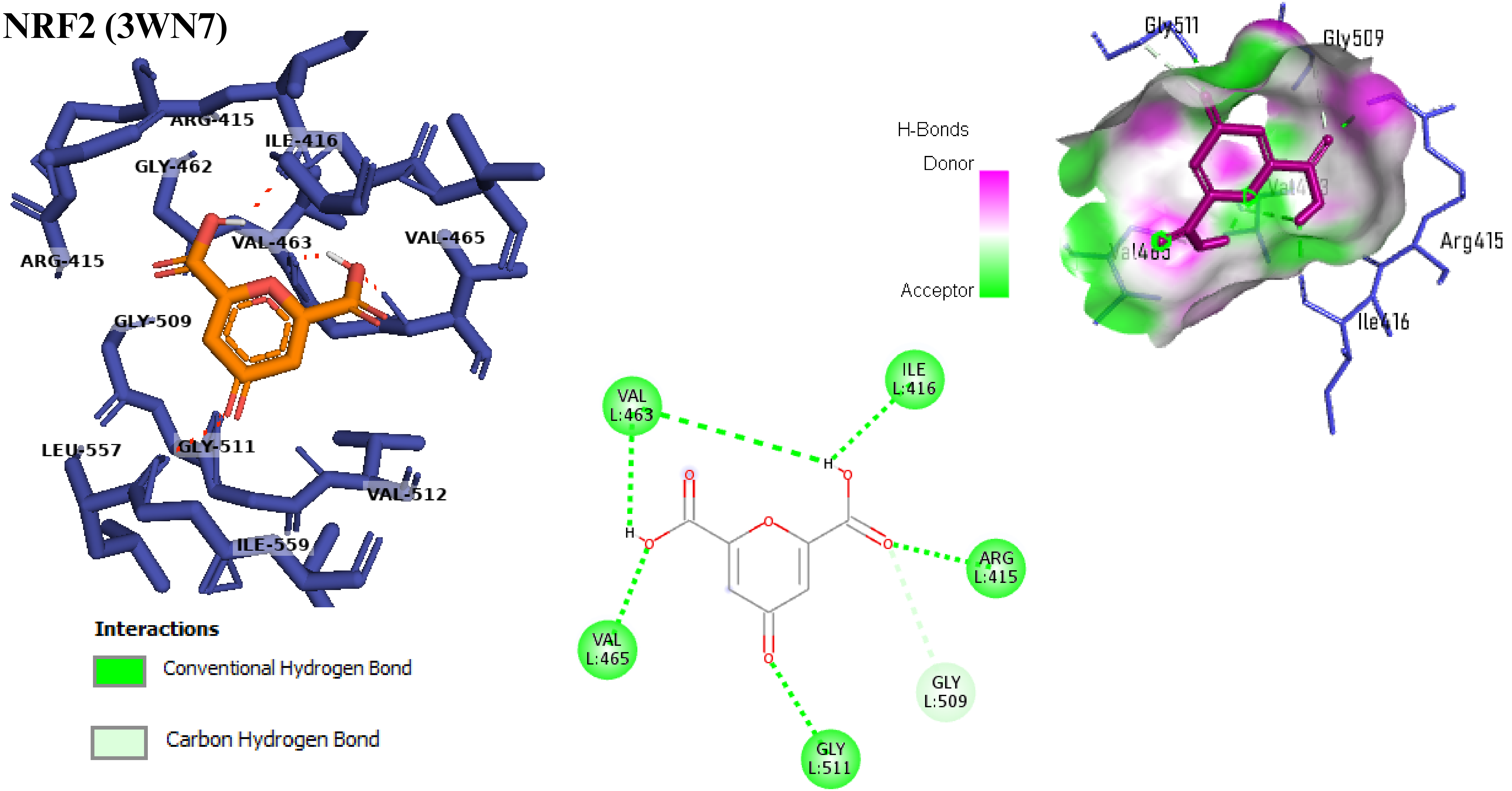

ARG-415
ILE-416
GLY-462
VAL-463
VAL-465
ARG-415
GLY-509
LEU-557
GLY-511
VAL-512
ILE-559
Interactions
Conventional Hydrogen Bond
Carbon Hydrogen Bond
VAL L:463
ILE L:416
VAL L:465
ARG L:415
GLY L:509
GLY L:511
H-Bonds
Donor
Acceptor
Gly511
Gly509
Arg415
Ile416

6. NF-kB (1SVC)

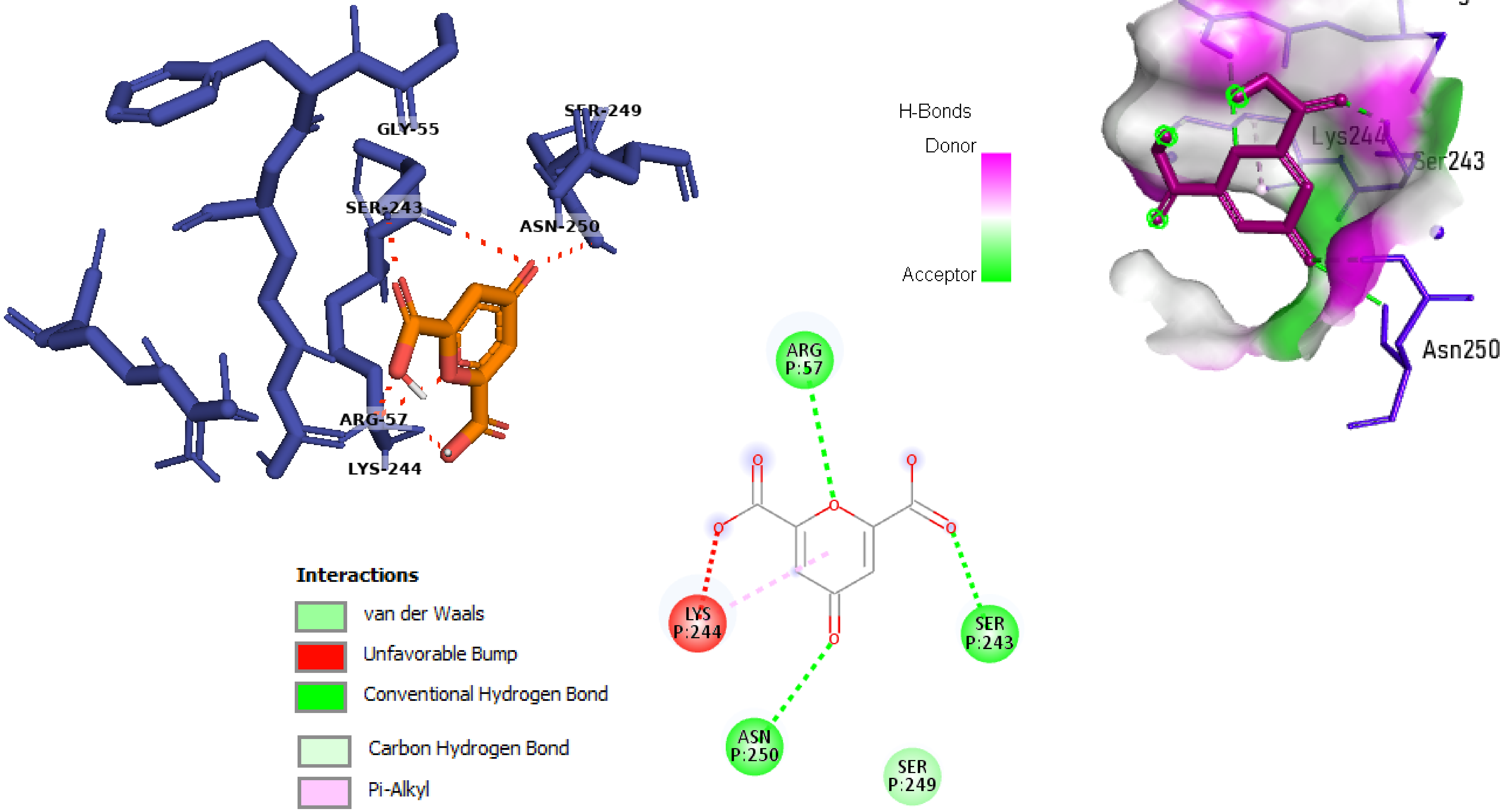

GLY-55
SER-249
SER-243
ASN-250
ARG-57
LYS-244
H-Bonds
Donor
Acceptor
Arg57
Lys244
Ser243
Asn250
ARG P:57
LYS P:244
SER P:243
ASN P:250
SER P:249
Interactions
van der Waals
Unfavorable Bump
Conventional Hydrogen Bond
Carbon Hydrogen Bond
Pi-Alkyl

7. JAK1 (4L00)

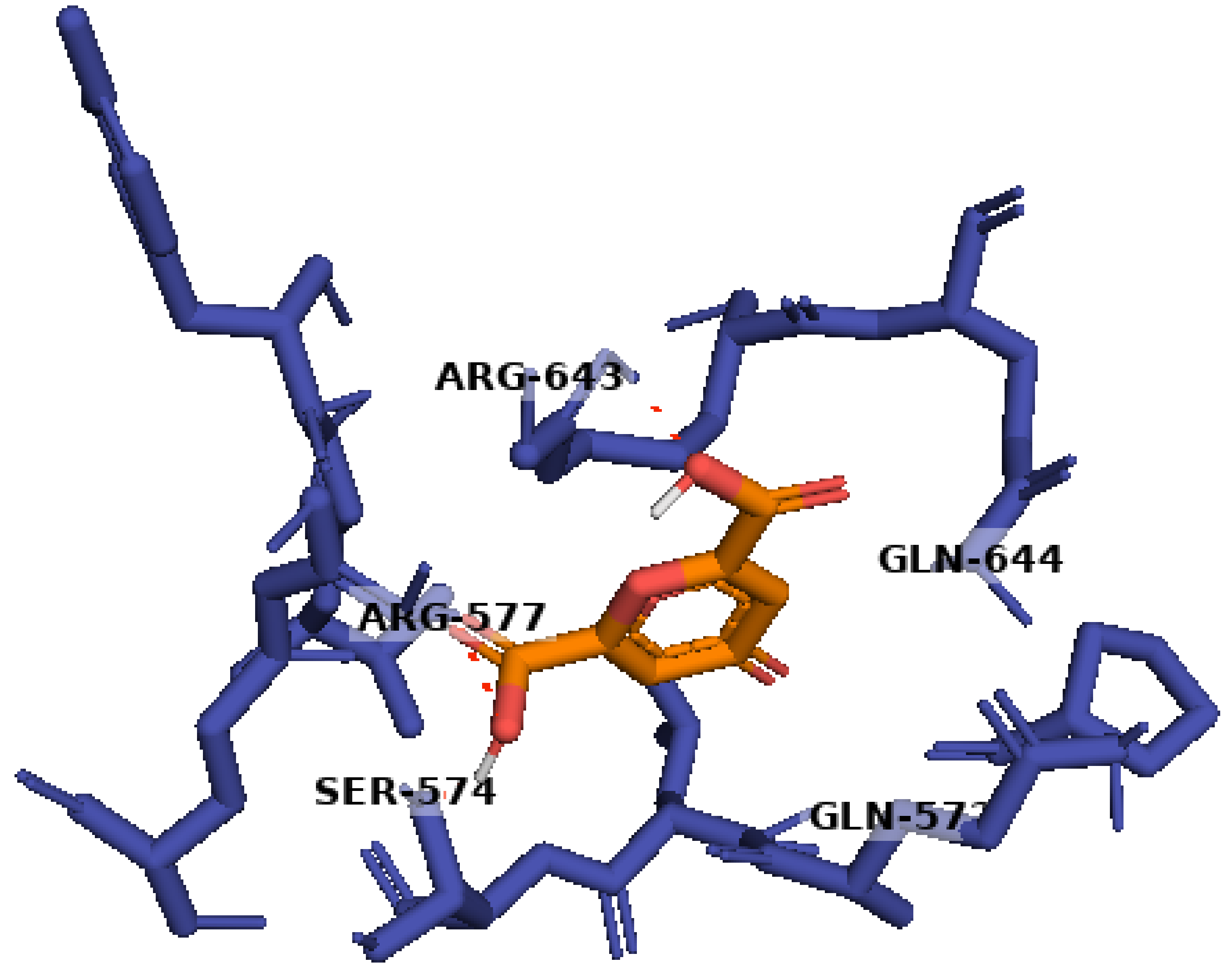
ARG-643
GLN-644
ARG-577
SER-574
GLN-57?

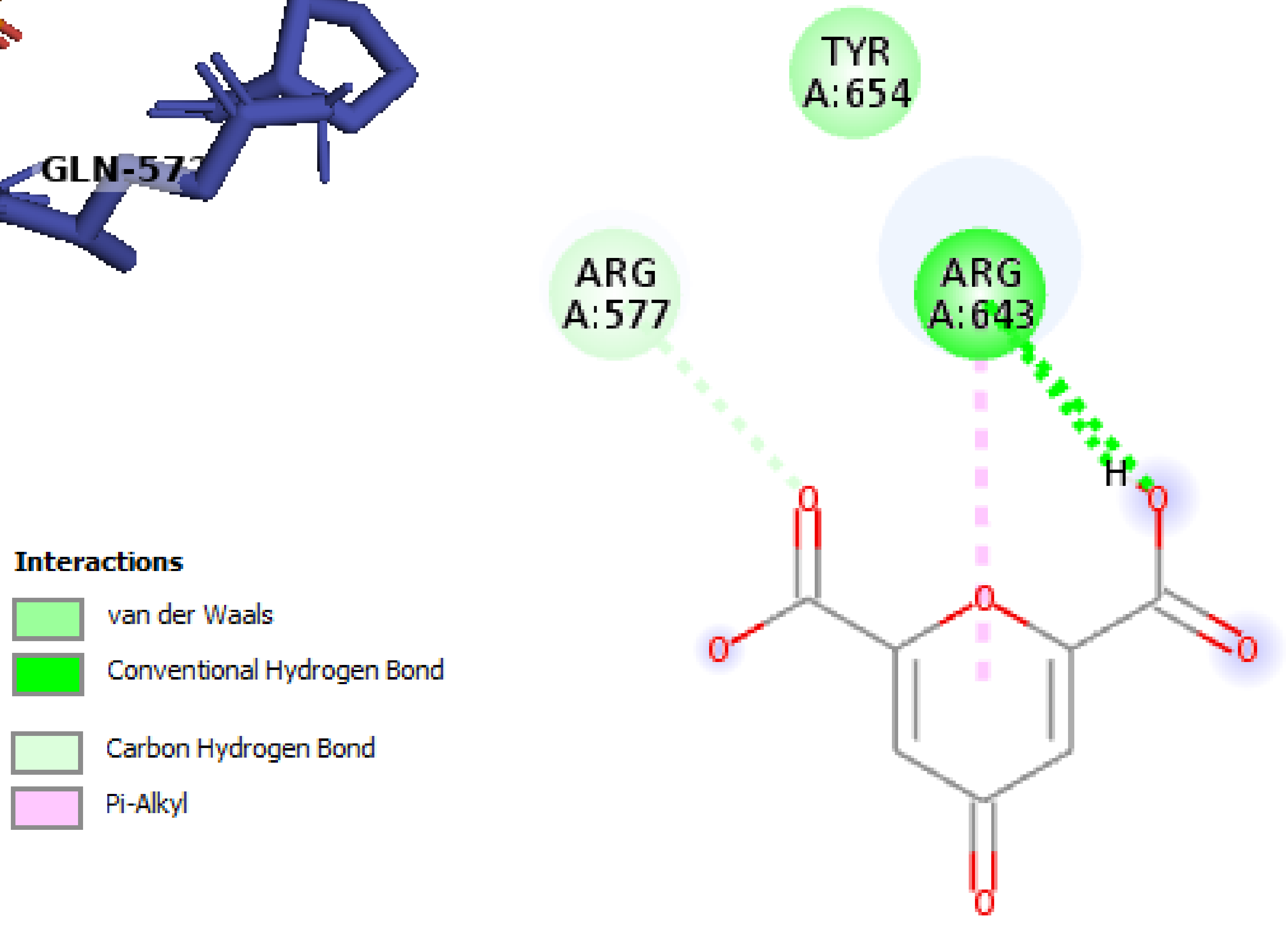
TYR
A:654
ARG
A:577
ARG
A:643
H
O
O
O
O
O
O
Interactions
van der Waals
Conventional Hydrogen Bond
Carbon Hydrogen Bond
Pi-Alkyl

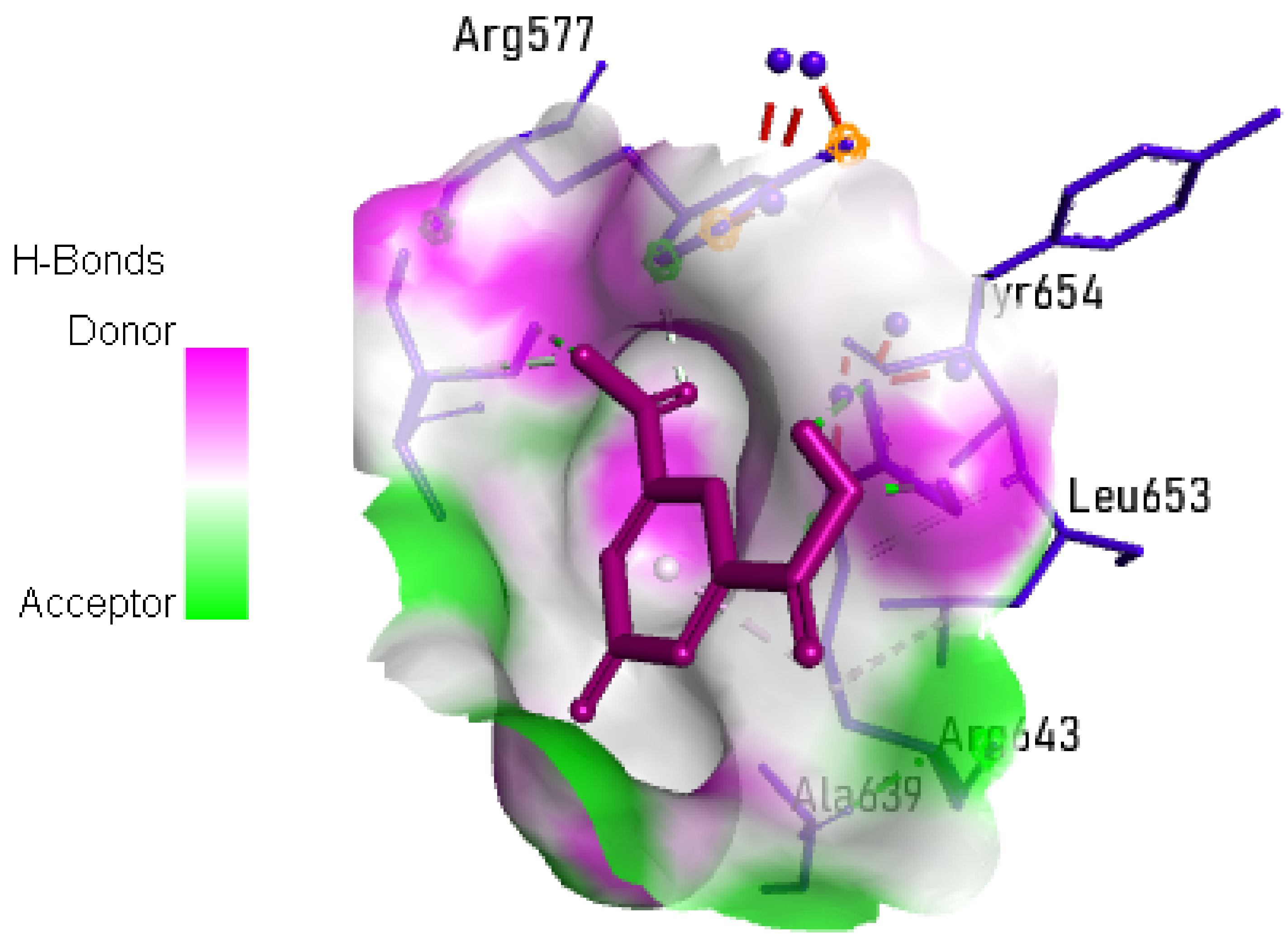
H-Bonds
Donor
Acceptor
Arg577
Leu653
Ala639

8.JAK2 (4GL9)

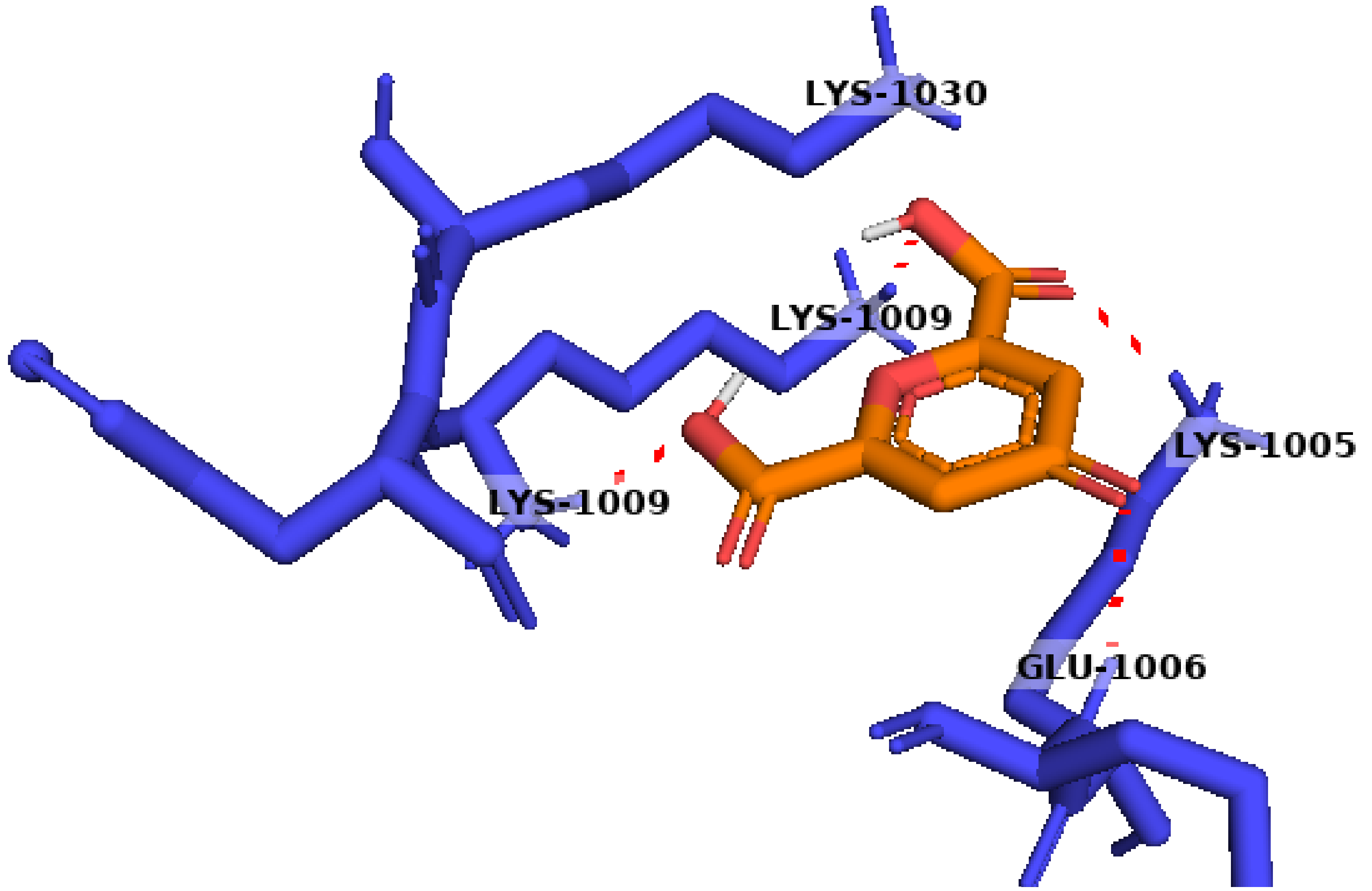
LYS-1030
LYS-1009
LYS-1009
LYS-1005
GLU-1006

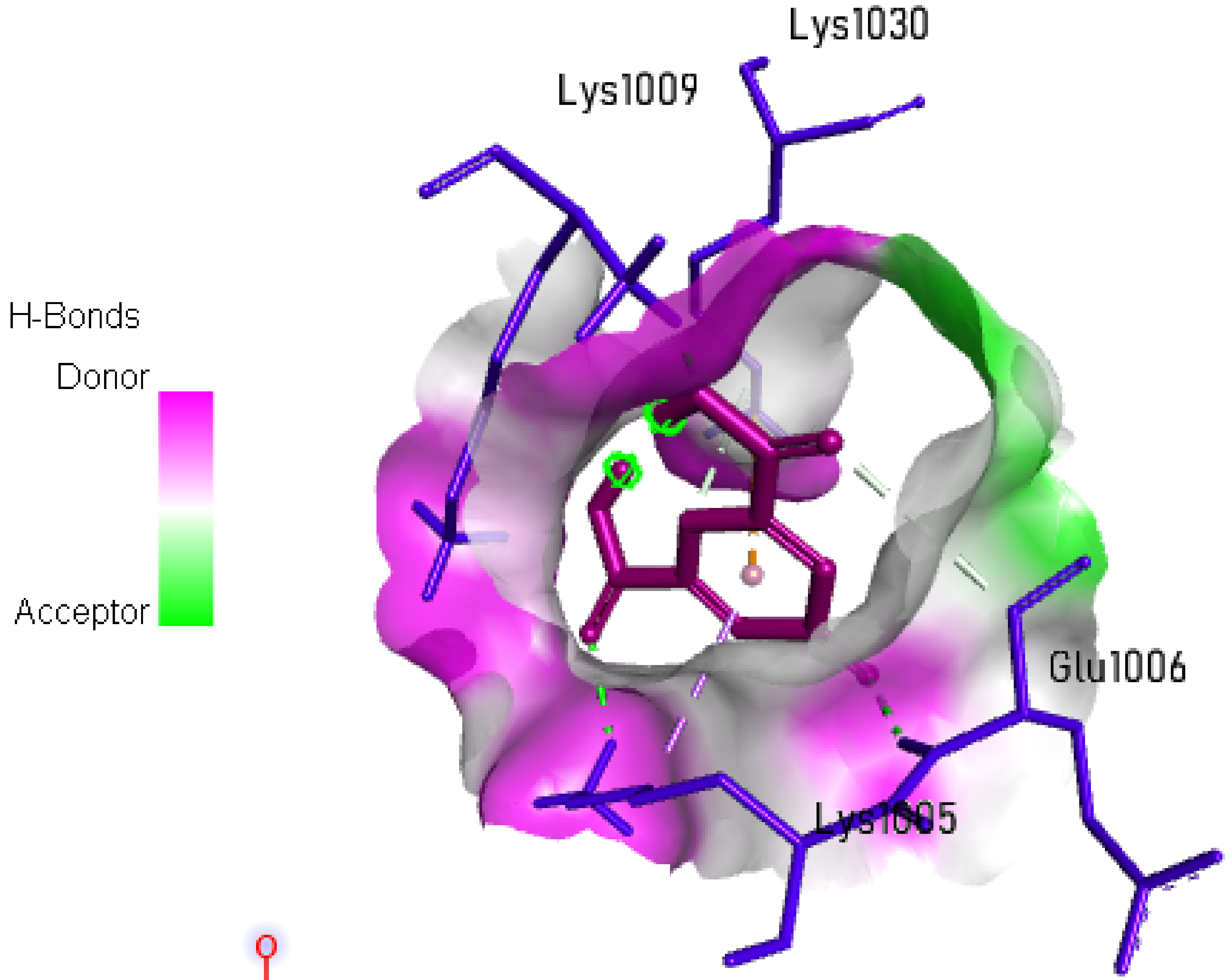
Lys1030
Lys1009
H-Bonds
Donor
Acceptor
Glu1006
Lys1005

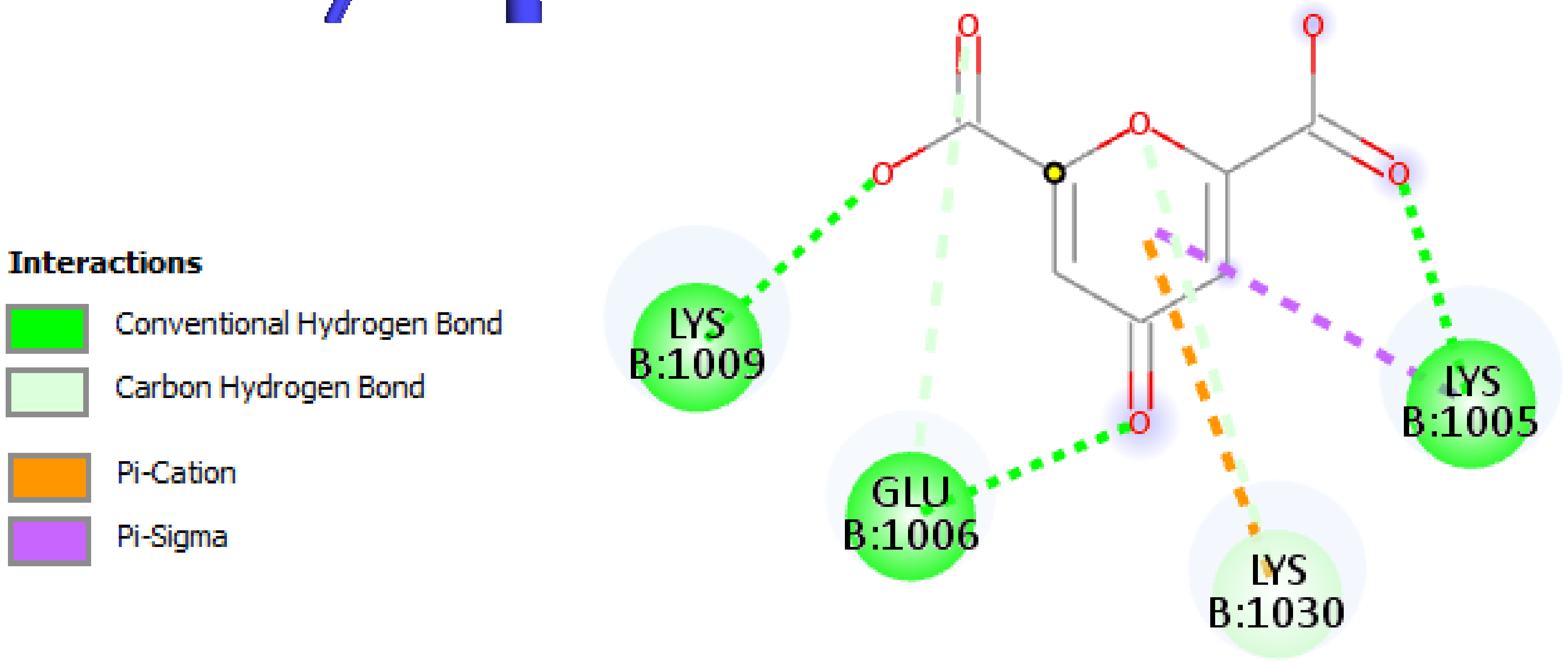
Interactions
Conventional Hydrogen Bond
Carbon Hydrogen Bond
Pi-Cation
Pi-Sigma
LYS B:1009
GLU B:1006
LYS B:1030
LYS B:1005

9. AMPK (7OPM)

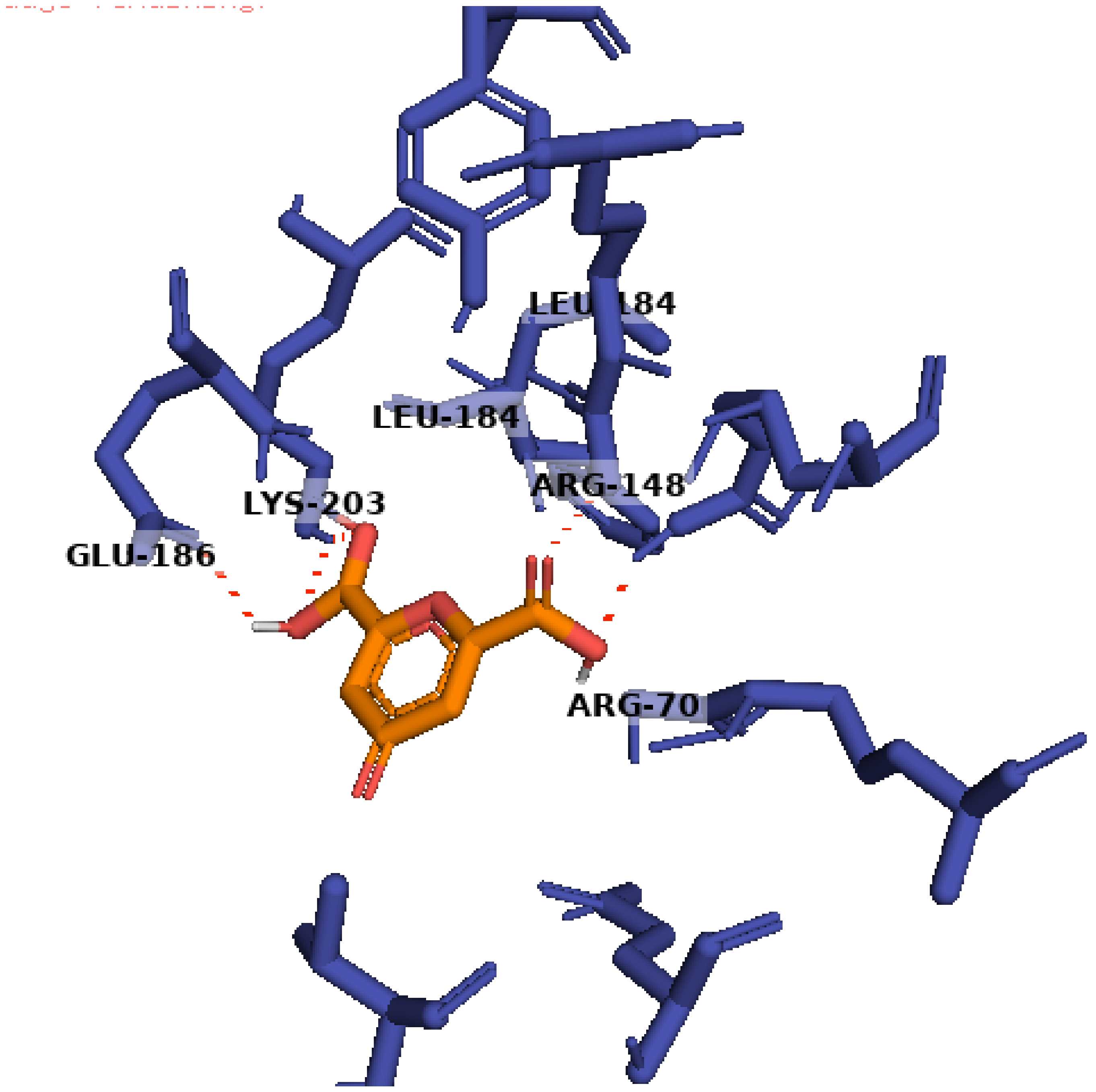
LEU-184
LEU-184
ARG-148
LYS-203
GLU-186
ARG-70

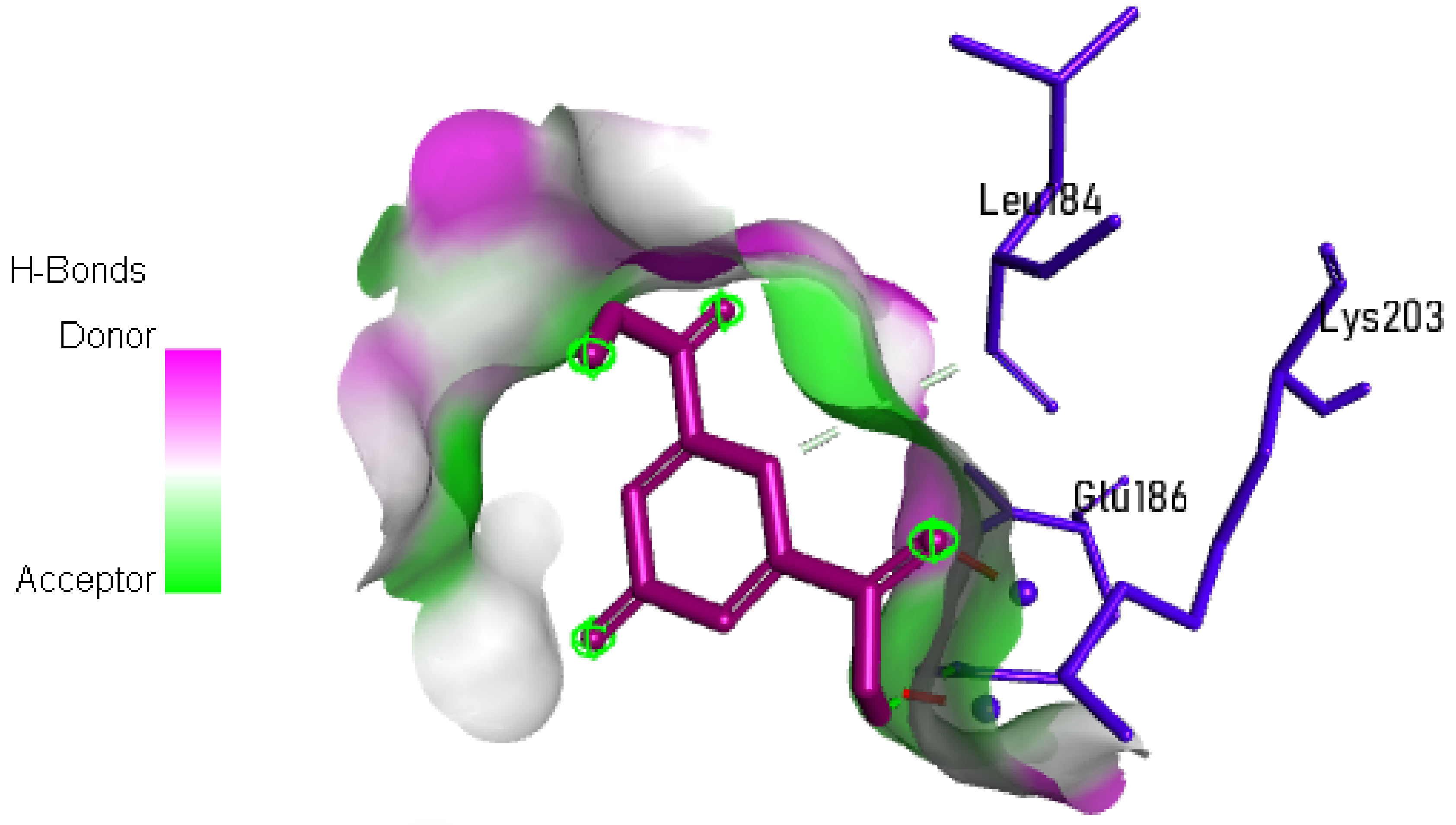
H-Bonds
Donor
Acceptor
Leu184
Lys203
Glu186

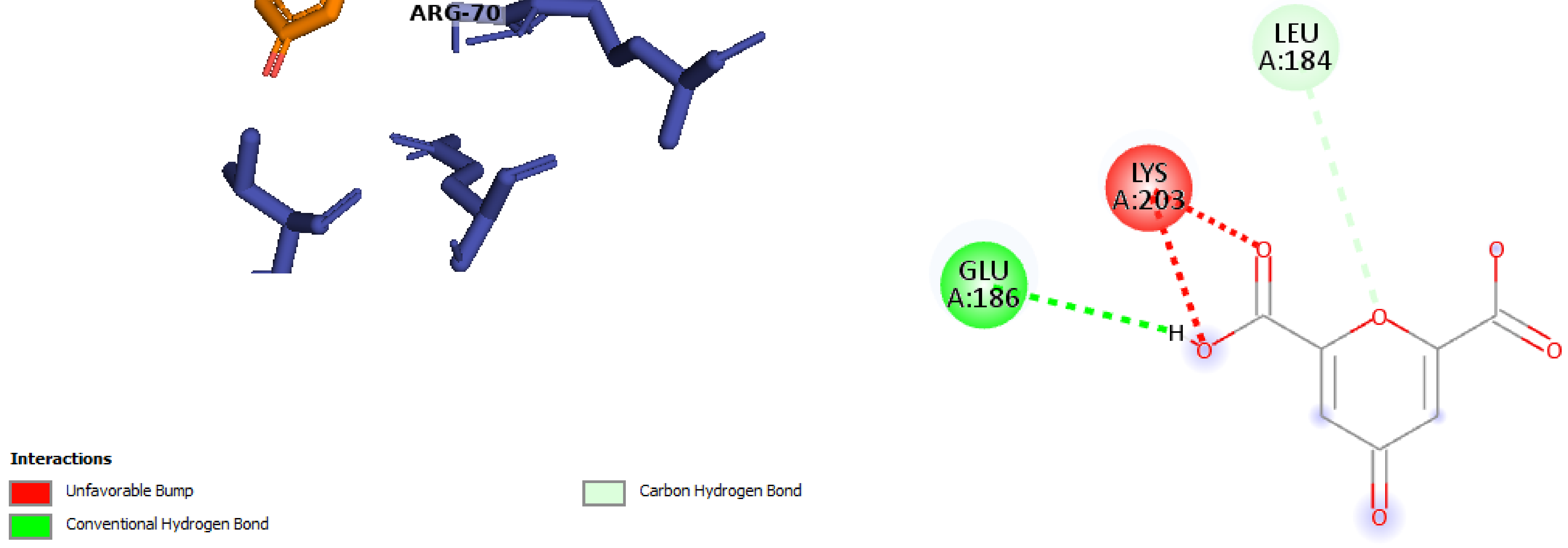
LEU
A:184
LYS
A:203
GLU
A:186
Interactions
Unfavorable Bump
Conventional Hydrogen Bond
Carbon Hydrogen Bond

10. STAT3 (5AX3)

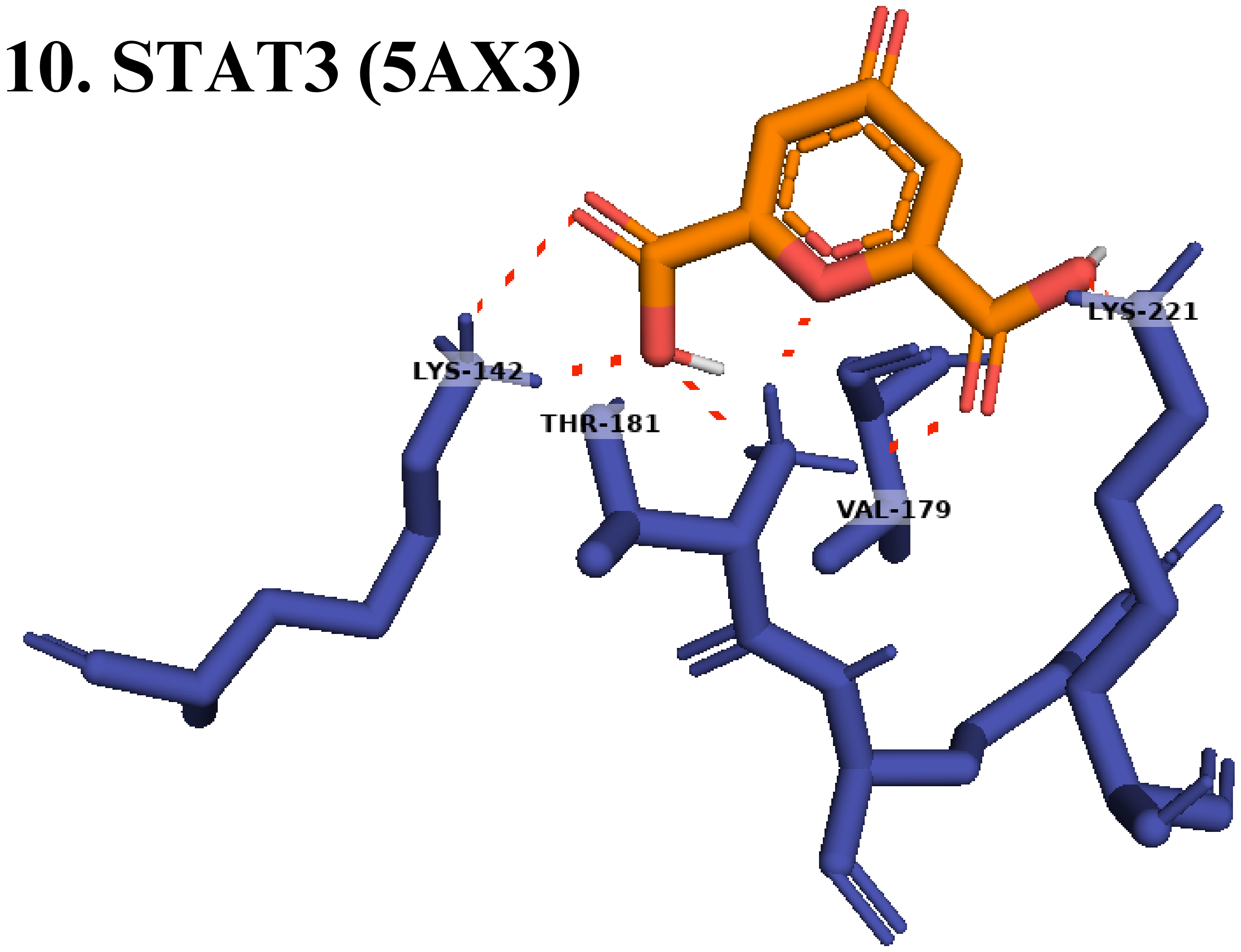

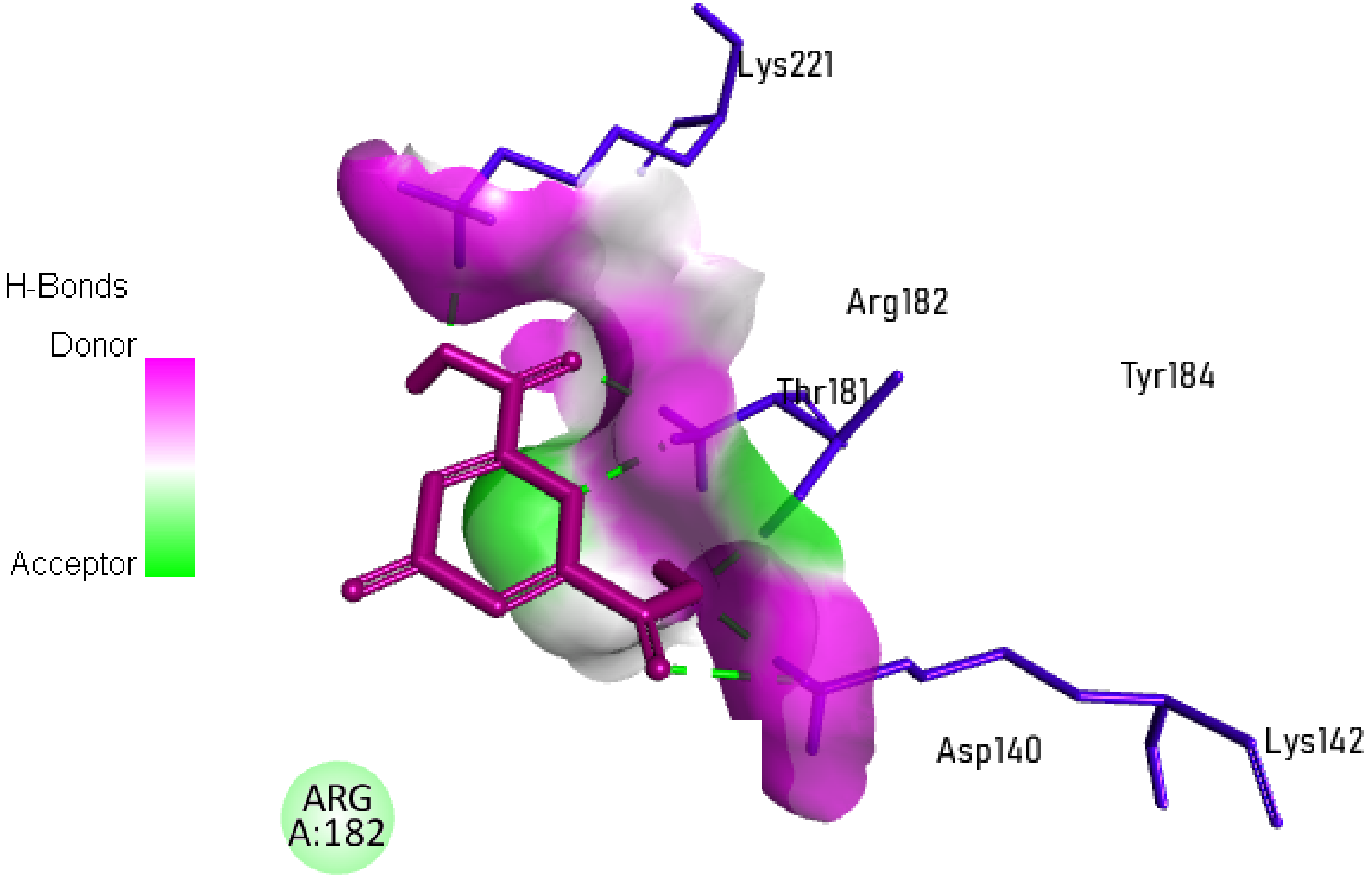

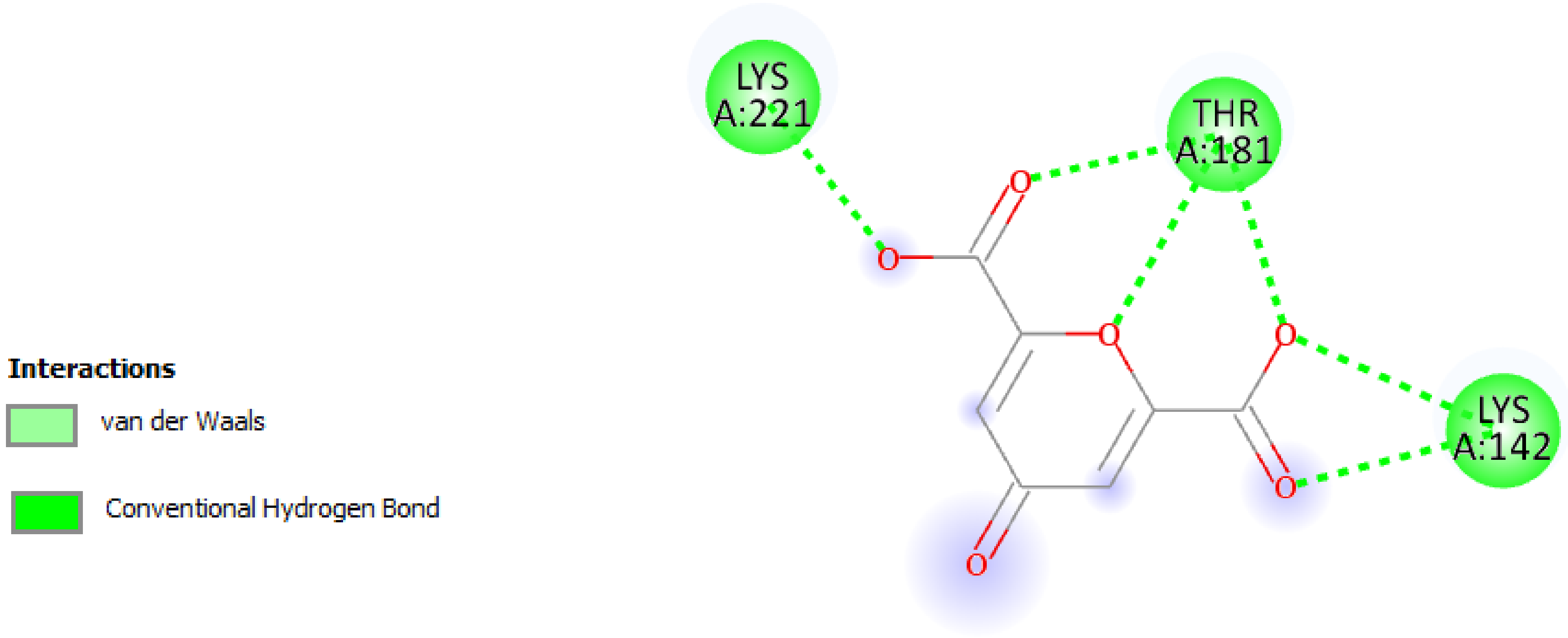

11.HSP90 (6KSQ)

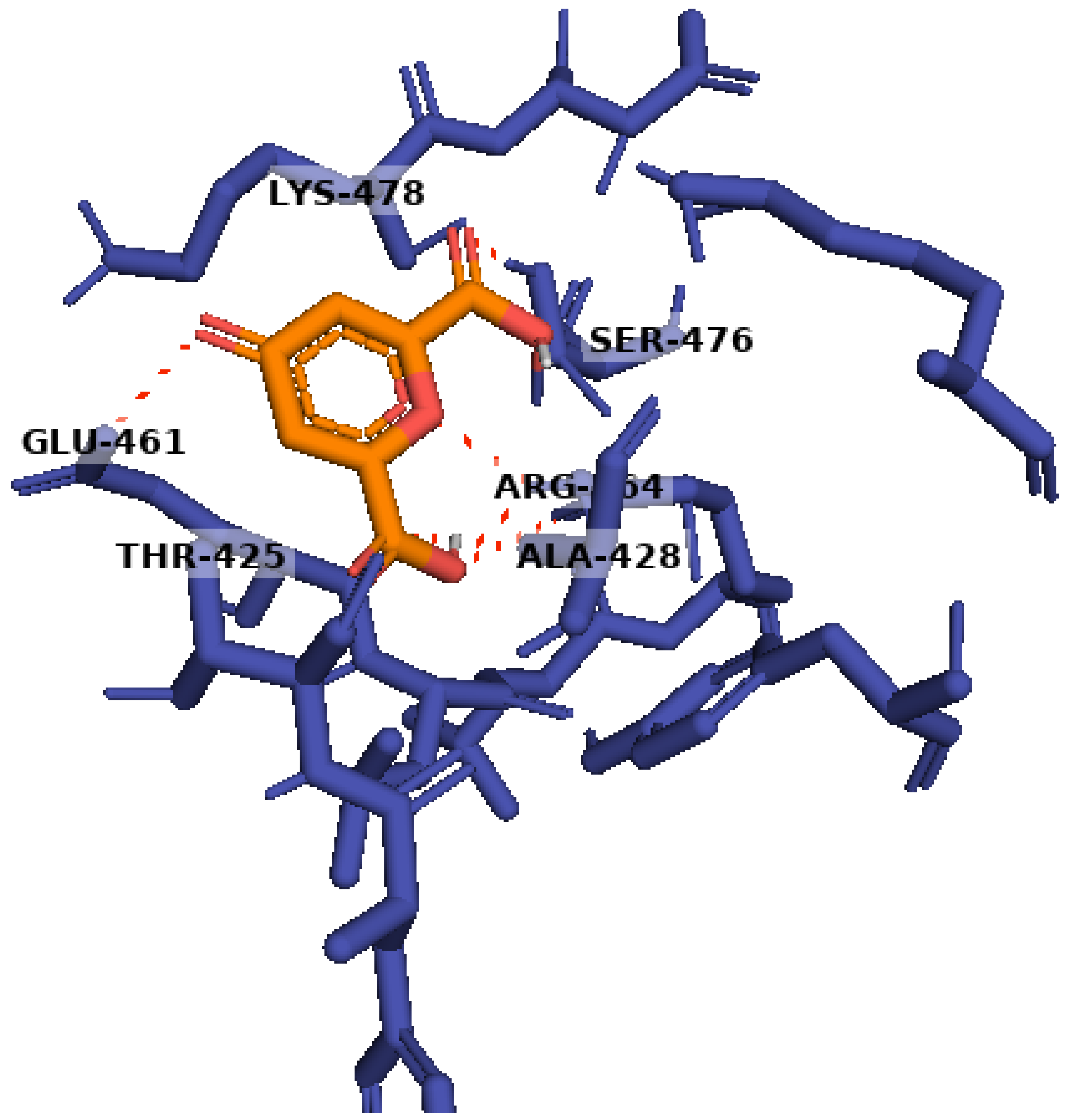

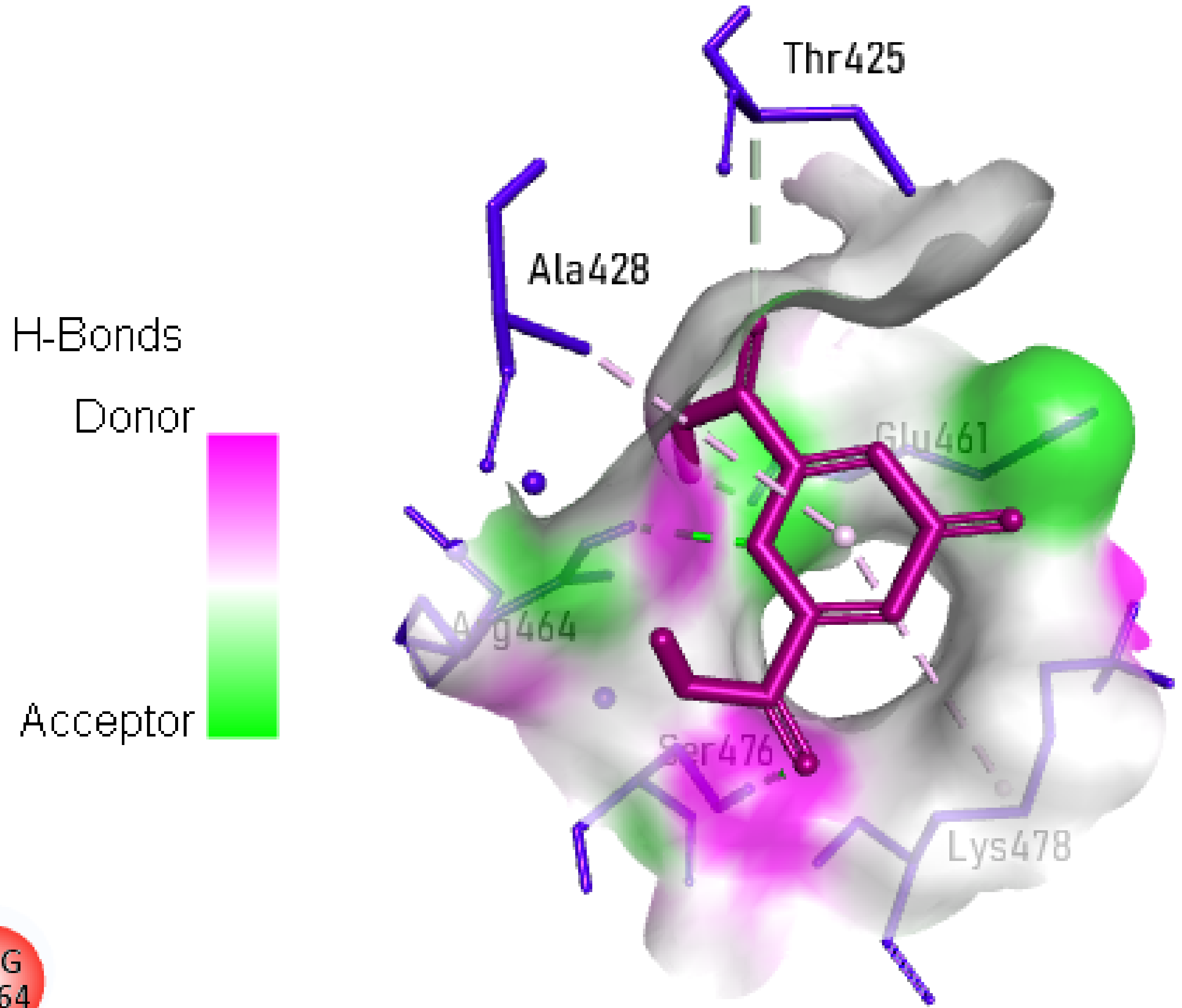

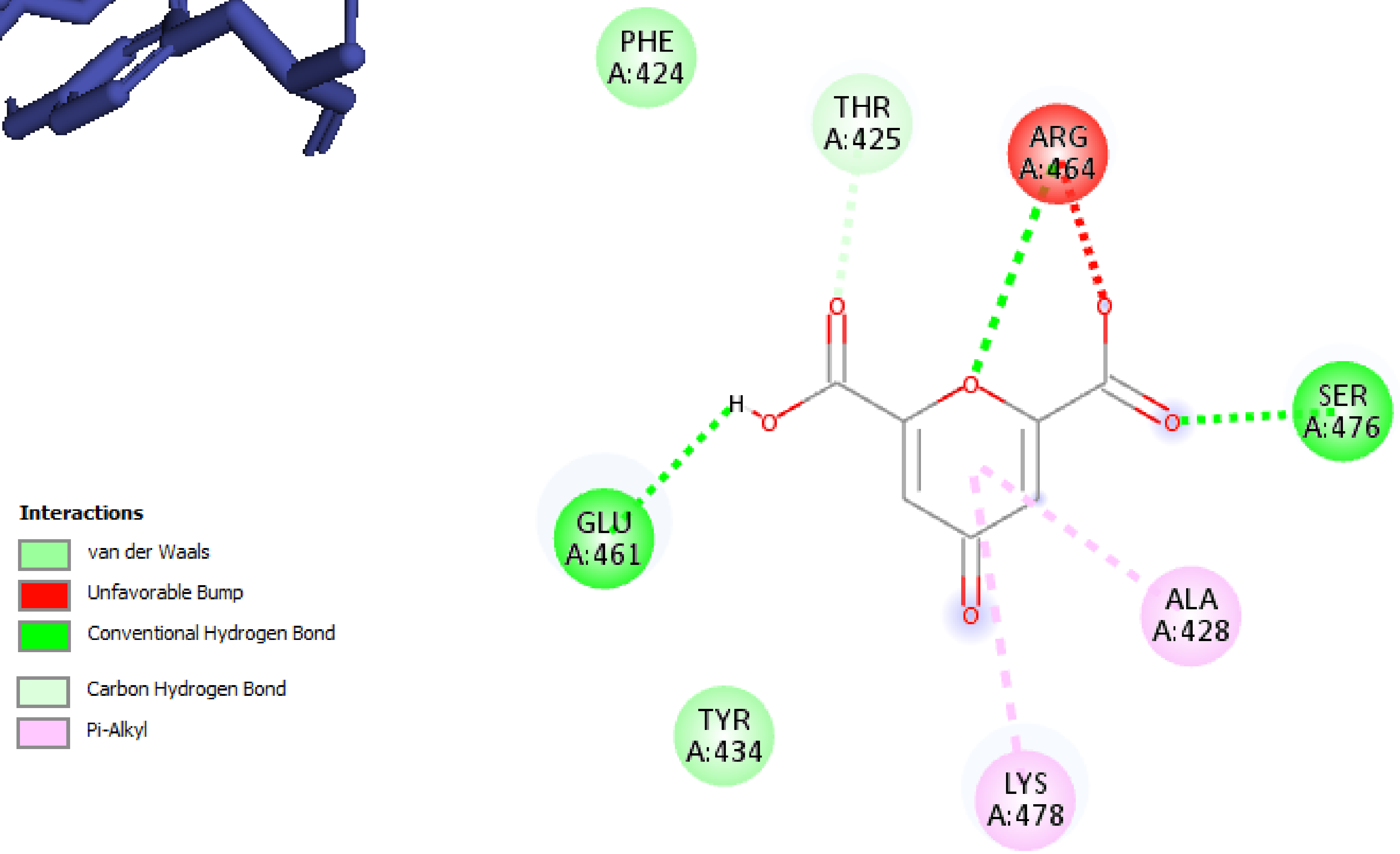

12.FOXO4 (3L2C)

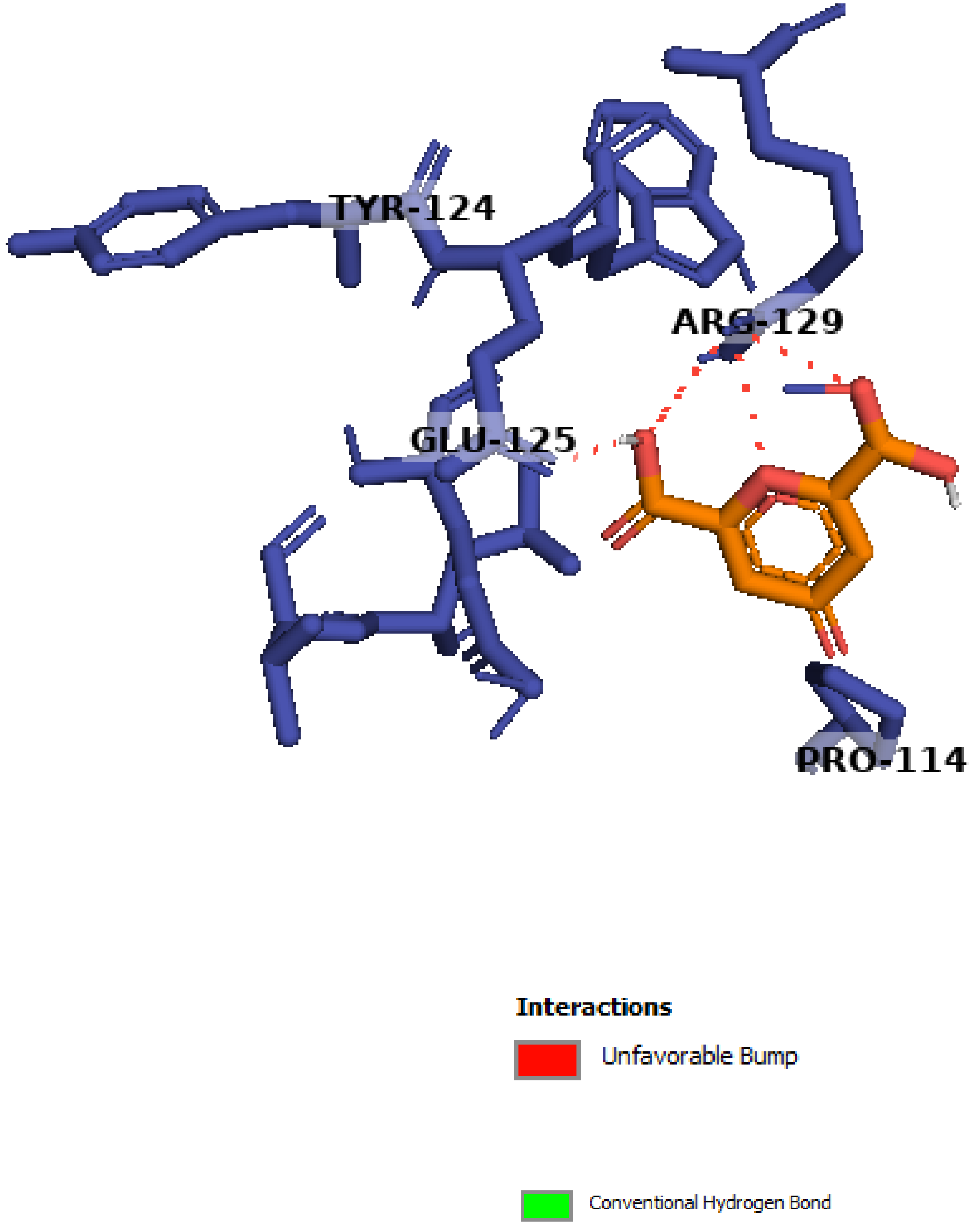

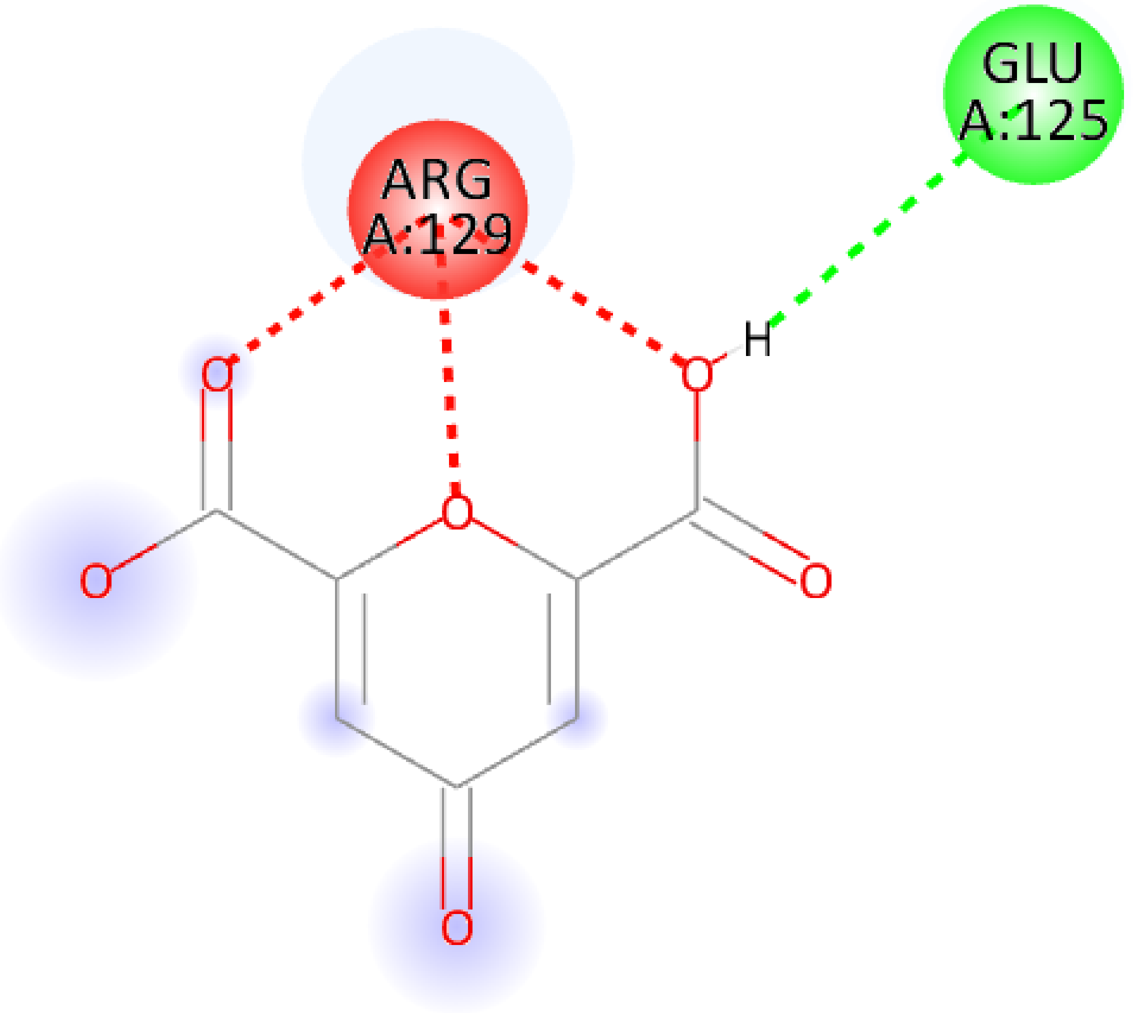

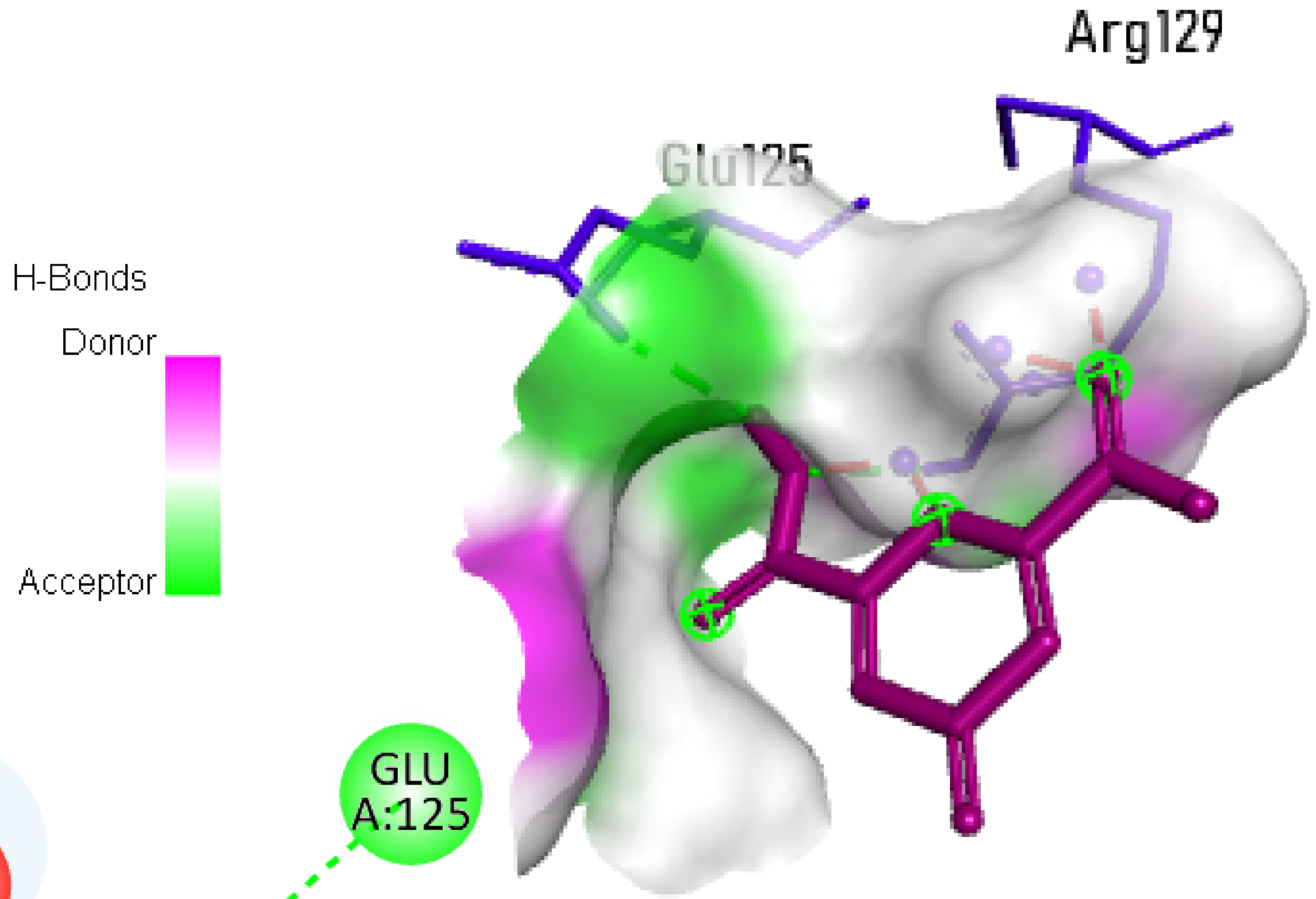

Supplement: Supplementary file 1 [file life-14-01070-s001.zip › Figure S1.pdf]
