# Supplementary material for: Investigating the Effects of Chelidonic Acid on Oxidative Stress-Induced Premature Cellular Senescence in Human Skin Fibroblast Cells
Source: Life (Basel). 2024 Aug 27;14(9):1070. doi: 10.3390/life14091070 (PMC11433492; doi:10.3390/life14091070)
Supplement: Supplementary file 1 [file life-14-01070-s001.zip › Table S1.pdf]

ID	Gene			%GC	Tm	Pair Base
M33647.1	**pRB1**	Forvard	TCACATTCCTCGAAGCCCTT	50	59.02	20
		Reverse	ACGGTCGCTGTTACATACCA	50	59.11	20
DQ263704.1	**p53**	Forvard	TGGCCATCTACAAGCAGTCA	50	59.02	20
		Reverse	GGTACAGTCAGAGCCAACCT	55	59.02	20
L27211.1	**p16**	forward	CCTCAGACATCCCCGATTGA	55	58.88	20
		reverse	GGACATTTACGGTAGTGGGG	55	57.68	20
BC000312.2	**p21**	forward	GGGCTGGGAGTAGTTGTCTT	55	59.02	20
		reverse	AGCCGAGAGAAAACAGTCCA	50	58.95	20
AF083106.2	**SIRT1**	forward	CCAGCCATCTCTCTGTCACA	55	59.1	20
		reverse	AACCTGTTCCAGCGTGTCTA	50	58.85	20
NM_001618.4	**PARP1**	forward	AGAAAAGGCGATGAGGTGGA	50	59.02	20
		reverse	AGCTCGTCCTTGATGTTCCA	50	59.02	20
U06454.1	**AMPK**	forward	GCGTCAAGGAATCCGAAGTC	55	59	20
		reverse	AGCTCGGTAAACTTCAGCCA	50	59.31	20
HM446346.1	**Nrf2**	forward	GGTTGCCCACATTCCCAAAT	50	59.02	20
		reverse	AGCAATGAAGACTGGGCTCT	50	59.01	20
AF488551.1	**APEX1**	forward	CCCTACCCAGCCCAGTTAAA	55	59.00	20
		reverse	CTCTCCCCACATTGTGTCCT	55	59.01	20
AY217036.1	**Bax**	forward	GATGACCCTCTGACCCTAGC	60	58.96	20
		reverse	CGGGCATTAAAGAGCTGGAC	55	58.98	20
JF919224.1	**CytochromeC**	forward	CAGGTAGGGGAGTTGGGATC	60	58.87	20
		reverse	TGTAAAGCCACCTCTCCCTG	55	59.02	20
NM_001101.5	**β-Actin (Control gene)**	forward	CCCTGGAGAAGAGCTACGAG	60	58.97	20
		reverse	CGTACAGGTCTTTGCGGATG	55	59	20
